# Single-cell RNA sequencing of mitotic-arrested prospermatogonia with *DAZL::GFP* chickens and revealing unique epigenetic reprogramming of chickens

**DOI:** 10.1186/s40104-022-00712-4

**Published:** 2022-06-06

**Authors:** Hyeon Jeong Choi, Kyung Min Jung, Deivendran Rengaraj, Kyung Youn Lee, Eunhui Yoo, Tae Hyun Kim, Jae Yong Han

**Affiliations:** 1 Department of Agricultural Biotechnology and Research Institute of Agriculture and Life Sciences, Seoul National University, Seoul, 08826 South Korea; 2 Department of Animal Science, Pennsylvania State University, State College, PA 16801 USA

**Keywords:** *DAZL::GFP* chickens, DNA methylation, Epigenetic reprogramming, Histone acetylation, Male germ cell, Mitotic arrest, Prospermatogonia, Single-cell RNA sequencing

## Abstract

**Background:**

Germ cell mitotic arrest is conserved in many vertebrates, including birds, although the time of entry or exit into quiescence phase differs. Mitotic arrest is essential for the normal differentiation of male germ cells into spermatogonia and accompanies epigenetic reprogramming and meiosis inhibition from embryonic development to post-hatch. However, mitotic arrest was not well studied in chickens because of the difficulty in obtaining pure germ cells from relevant developmental stage.

**Results:**

We performed single-cell RNA sequencing to investigate transcriptional dynamics of male germ cells during mitotic arrest in *DAZL*::*GFP* chickens. Using differentially expressed gene analysis and K-means clustering to analyze cells at different developmental stages (E12, E16, and hatch), we found that metabolic and signaling pathways were regulated, and that the epigenome was reprogrammed during mitotic arrest. In particular, we found that histone H3K9 and H3K14 acetylation (by *HDAC2*) and DNA demethylation (by *DNMT3B* and *HELLS*) led to a transcriptionally permissive chromatin state. Furthermore, we found that global DNA demethylation occurred gradually after the onset of mitotic arrest, indicating that the epigenetic-reprogramming schedule of the chicken genome differs from that of the mammalian genome. DNA hypomethylation persisted after hatching, and methylation was slowly re-established 3 weeks later.

**Conclusions:**

We found a unique epigenetic-reprogramming schedule of mitotic-arrested chicken prospermatogonia and prolonged hypomethylation after hatching. This will provide a foundation for understanding the process of germ-cell epigenetic regulation in several species for which this process is not clearly described. Our findings on the biological processes related to sex-specific differentiation of prospermatogonia could help studying germline development in vitro more elaborately.

**Supplementary Information:**

The online version contains supplementary material available at 10.1186/s40104-022-00712-4.

## Background

Primordial germ cells (PGCs) are precursors of ova and sperm, which carry genetic information to the next generation [1]. PGC development, including specification, migration, proliferation, and differentiation, is mainly regulated in a time-dependent manner [2]. During differentiation of bipotential PGCs into sex-specific germ cells in birds and mammals, female germ cells continue to proliferate and enter meiosis before birth, whereas male germ cells enter mitotic arrest [3–5]. We previously reported that chicken female germ cells enter meiosis from embryonic day (E) 13 and maintain meiotic arrest in G_2_/M phase until hatch. But chicken male germ cells asynchronously enter mitotic arrest from E14 and remain in G_0_/G_1_ phase until hatch [6]. Mitotic arrest is a prominent event in prospermatogonia (which is also known as gonocyte and is the pre-stage of spermatogonia), and is necessary for proper differentiation of male germ cells [7]. Defects in sex-specific differentiation of male germ cells can lead to either abnormal entry into mitotic arrest or abnormal re-entry into mitosis [8–10].

Studies of cell-cycle genes regulating mitotic arrest showed that increased Cip/Kip and INK4 inhibitors and modulation of retinoblastoma 1 cause mitotic arrest in mouse germ cells [3, 11]. Normal mitotic arrest fails during teratoma formation in the testicular-teratoma-sensitive mouse-strain 129/SvJ. Gene mapping in this mutant mouse strain revealed that dead end homolog 1 regulates mitotic arrest upstream of cell-cycle-regulatory genes [12]. A study of zinc-finger protein basonuclin 2-deletion mice showed that prospermatogonia entered mitotic arrest late, underwent abnormal meiosis, and failed to form pools of spermatogonial stem cells [8]. In addition, studies inhibiting Sertoli cell proliferation in mice reported that prospermatogonia to escape from mitotic arrest and re-enter mitosis, resulting in higher levels of apoptosis and infertility [9, 13]. Prospermatogonia mitotic arrest is conserved in a variety of species, including human, rodent, cattle, and tammar wallaby [14–16], although the time scales of arrest between species differ [17]. However, studies of this quiescence phase in species other than mouse are very limited. Single-cell RNA-sequencing (scRNA-seq) studies recently revealed transcriptional features of mitotically quiescent prospermatogonia in humans [18]. This understanding has been applied both to in vitro cultures and to organogenesis [19, 20].

Epigenetic reprogramming of male germ cells during embryonic development involves DNA demethylation to erase the paternal imprint and to allow proper PGC differentiation; then, re-methylation, to establish paternal imprinting and to repress transposable elements [21–24]. In other words, DNA re-methylation is also a concomitant process in mitotic-arrested prospermatogonia. In mice, DNA demethylation occurs when PGCs migrate to the genital ridge. DNA is then remethylated (with higher *Dnmt3a* and *Dnmt3l* expression levels in the developing testis) shortly after initiation of mitotic arrest [25]. In humans, DNA demethylation of PGCs occurs alongside repression of UHRF1, DNMT3A, and DNMT3B, and most imprints are erased before reaching the genital ridge (thus occurring earlier than in mice) [26, 27]. Human PGCs DNA methylation levels are also lowest before entering mitotic arrest. However, the process of human PGCs re-methylation is still unclear [27, 28]. Chicken PGCs are detected at the Eyal-Giladi and Kochav (EGK) stage-III [1], and the first epigenetic event in chicken germ cells is DNA demethylation, which occurs as PGCs migrate from the germinal crescent to extra-embryonic blood vessels [29]. Our previous study demonstrated that chicken gonadal PGCs (E4.5 and E6.5) have higher global 5-methylcytosine (5-mC) levels than do blood PGCs (E2.5) [30]. Also, He et al. showed that DNA methylation levels are lower in chicken embryo spermatogonial stem cells isolated at E19 than in PGCs isolated at E5.0 [31]. Therefore, de novo methylation is established during germ cell migration through blood vessels to the genital ridge, and demethylation occurs again in the chicken male germline.

During the transition of PGCs to gonocytes in mouse, loss of global DNA demethylation, polycomb repressive complex 1 (PRC1)-mediated repression, and Tet1 recruitment are required to erase imprinting and to increase germline reprogramming-responsive gene activity, which enable gametogenesis [23]. A recent study using scRNA-seq and bisulfite sequencing in mouse revealed that only demethylated germ cell clusters differentiate into male germ cells that further express germline reprogramming-responsive genes. Germ cell clusters with aberrant epigenetic reprogramming are instead directed toward apoptosis [23, 32]. Moreover, a study on how mitotically arrested prospermatogonia facilitate de novo DNA methylation suggested that differentially accessible domains form before de novo DNA methylation and are recruited by histone modifiers [33]. As such, epigenetic reprogramming plays a key role in ensuring proper differentiation into spermatogonia, and there is much interest in epigenetic dynamics of mitotic-arrested spermatogonia [7, 28].

Studies of male germ cells after sex determination in chickens are limited because we lack antibodies to isolate living cells. However, we recently produced *DAZL::GFP* chickens with a green fluorescent protein (*GFP*) reporter inserted into germ cell-specific deleted in azoospermia like (*DAZL*) gene using CRISPR/Cas9- nonhomologous end joining (NHEJ)-mediated genome editing technology, which enabled us to trace and efficiently isolate germ cells at all developmental stages. In this study, we investigated differentiation of male germ cells from *DAZL::GFP* chickens by using scRNA-seq. We isolated germ cells at the three mitotic arrest-related time points: E12, mitotically active germ cells; E16, germ cells entering mitotic arrest asynchronously; hatch, mostly mitotic-arrested prospermatogonia [6]. We reveal distinct transcriptional changes during the transitions before and after the onset of mitotic arrest and uncover unique epigenetic reprogramming patterns. We show significant changes in expression of *HDAC2*, *DNMT3B*, and *HELLS*, confirming higher histone acetylation levels and global DNA demethylation in mitotic-arrested prospermatogonia. Unlike mouse and pig prospermatogonia, which rapidly remethylate, mitotic-arrested prospermatogonia in chickens remethylate slowly only 3 weeks post-hatch. These results advance our understanding of gamete formation and of epigenetic reprogramming patterns after the onset of mitotic arrest.

## Methods

### Experimental animals and animal care

The care and experimental use of chickens was approved by the Institute of Laboratory Animal Resources, Seoul National University (SNU-190401-1-1 and SNU-190401-1-2). All procedures, including chicken maintenance, reproduction, and sample collection, were governed by standard operating protocols according to a standard management program at the University Animal Farm, Seoul National University and the Animal Genetic Engineering Laboratory at Seoul National University.

### Sample preparation for single-cell RNA sequencing from *DAZL::GFP* chickens

To isolate the stage-specific germ cells used in this study, we established *DAZL::GFP* chickens through CRISPR/Cas9-NHEJ-mediated genome editing system. According to the methods and procedures described previously [34], we constructed all-in-one CRISPR/Cas9 plasmids targeting the last intron of the *DAZL* gene, and we synthesised the last intron of *DAZL* including the gRNA recognition sequence and the last exon in frame with a T2A peptide and GFP expression cassette were constructed (Bioneer, Daejeon, Korea) as a donor plasmid. The donor plasmids (2 μg) and CRISPR/Cas9 plasmids (2 μg) were co-introduced into 1 × 10^5^ cultured PGCs with 4 μL of Lipofectamine 2000 reagent (Thermo Fisher–Invitrogen, Carlsbad, CA, USA) suspended in 1 mL of Opti-MEM, and neomycin selection was conducted for 3 weeks. To produce genome-edited chickens, a window was cut at the sharp end of the Hamburger and Hamilton (HH) stages 14–17 Korean Ogye-breed recipient egg (*i*/*i* gene, black feather color), and more than 3000 DAZL::GFP tagged genome-edited White Leghorn (WL) PGCs (*I*/*I* gene, white feather color) were transplanted into the dorsal aorta of HH stages 14–17 recipient embryos (*i*/*i*). The egg window was sealed with paraffin film and the eggs were incubated with the pointed end down until hatching. After sexual maturation, sperms from the recipient chickens were evaluated by breed- specific PCR conditions, and the chickens that had WL sperm were mated with WL wild-type female chickens (*I*/*I*). Sine WL chicken has a dominant pigmentation inhibitor gene (*I/I*), while Korean Ogye has a recessive pigmentation inhibitor gene (*i/i*), feather-colored dominance is commonly used for the easy identification of donor PGC-derived offspring [35]. Genome-edited chickens (*I*/*I*) were identified based on feather color and subsequent genomic DNA analysis.

DAZL::GFP germ cells at mitotic arrest-related three time points were collected from testes (at E12, E16 and hatch). The testes were microscopically dissected and pooled from 10 male embryos or chicks at E12, E16, and hatch, respectively (Additional file 1: Table S1). The pooled testes were treated with Hank’s Balanced Salt Solution (HBSS) containing 0.05% trypsin-EDTA (Gibco, Waltham, MA, USA) and incubated at 37 °C for 10 min. Every 2 min, pipetting was conducted during incubation, and after finishing the incubation, trypsin EDTA solution was inactivated by the same volume of Dulbecco’s minimum essential medium (DMEM) containing 5% fetal bovine serum (FBS). The cell suspension was harvested by centrifugation (1250 r/min, 5 min), and washed with cold phosphate-buffered saline (PBS). The cells were suspended with PBS containing 1% bovine serum albumin (BSA) and filtered through 40 μm cell strainer (Falcon™ 352340, Fisher Scientific, Hampton, NH, USA). To isolate live cells, cells were stained with propidium iodide (PI), and GFP^+^/PI^−^ cells were sorted by using a BD fluorescence-activated cell sorting (FACS) Aria III (BD Biosciences, San Jose, CA, USA). Information on the number of cells sorted by FACS is listed in Additional file 1: Table S1.

Additionally, DAZL::GFP germ cells at E2.5, E6, E8, and 1 week post-hatch were collected to observe the expression patterns of DNA methylation-related and mitosis-related genes from blood-circulating PGCs up to 1 week after hatching. Whole blood cells were isolated from the dorsal aorta of each embryo using a glass micropipette under a microscope from 70 to 80 embryos at E2.5 [36]. The gonads or testes were microscopically dissected and pooled from 35 embryos, 11 embryos, and 3 chicks at E6, E8, and 1 week post-hatch, respectively (Additional file 1: Table S1). The sex of E2.5, E6, and E8 embryos was determined (at E2.5) by sex-discriminating PCR of blood samples [30]. The sex of E2.5 germ cell was further confirmed during scRNA-seq data preprocessing on the basis of W-chromosome gene expression. Signature scores of the genes on W chromosome were calculated and cluster with negative score were annotated as male PGCs. The pooled gonads at E6 and E8 and the pooled testes at 1 week post-hatch were treated to dissociate to single cells in the same manner as above. Then the samples were stained with PI as described above, and GFP^+^/PI^−^ cells were sorted by BD FACS Aria III at E2.5, E6, E8, and 1 week post-hatch. Information on the number of cells sorted by FACS is listed in Additional file 1: Table S1.

### Single-cell RNA sequencing

Libraries for scRNA-seq were prepared by using the Chromium Single Cell 3′ GEM, Library & Gel Bead Kit v3 (PN-1000075, 10 × Genomics, Pleasanton, CA, USA); Chromium Single Cell B Chip Kit (PN-1000073, 10 × Genomics); and Chromium i7 Multiplex Kit (PN-120262, 10 × Genomics). Cells were resuspended in PBS containing 0.04% BSA and diluted to ~ 2 × 10^5^ to ~ 1 × 10^6^ cells/mL. Cells were mixed with a reverse-transcription master mix and loaded onto B chip channels to capture ~ 800 to ~ 5000 single-cell transcriptomes. Gel bead-in emulsions (GEMs) were generated by using Chromium Controller (10 × Genomics). Reverse transcription was conducted by using a C1000 Touch thermal cycler (Bio-Rad, Hercules, CA, USA). DNA was purified, and libraries were constructed according to the manufacturer’s instruction. The qualities of amplified cDNAs and of the constructed libraries were assessed by using Bioanalyzer (Agilent Technologies, Santa Clara, CA, USA). Libraries were sequenced with a 2 × 100-bp paired-end protocol on a Novaseq-6000 platform (Illumina, San Diego, CA, USA) to generate at least 40,000 read pairs per cell. The sequencing depth recommended by the manufacturer for the 3′ Gene Expression library is a minimum of 20,000 read pairs per cell, and values in the range of 20,000 to 50,000 read pairs per cell are commonly used in the field [37, 38].

### Single-cell RNA-seq data processing and analysis

Raw fastq files were processed using the CellRanger pipeline, version 3.1.0. The fasta and GTF files for chicken genome (GRCg6a) were modified to include the *DAZL*-*GFP* insert sequence. The cDNA sequences were mapped to the modified-chicken genome by using STAR, version 2.5.1b, aligner [39] with the GRCg6a.99 GTF file. A gene-by-cell count matrix was generated by using default parameters. To remove empty droplets while capturing single cells, the EmptyDrops function of DropletUtils, version 1.8.0, R package [40] was used (with FDR < 0.05). We diagnosed low-quality cells by visualizing features such as the number of unique molecular identifier (UMI), the number of detected genes, and proportion of the mitochondrial gene count generally used for quality control (QC) [41, 42]. Low-quality cells were excluded by using different cutoff thresholds for different samples. The cutoff thresholds were determined by visually inspecting outliers in the principal component analysis (PCA) plot on the quality-control metrics using the calculateQCMetrics function of the scater, version 1.16.1, R package [43]. The values used for the QC criteria for each sample are depicted in Additional file 2: Fig. S1A, and several PCA plots are shown in Additional file 2: Fig. S1B. The number of cells remaining after QC is shown in Additional file 1: Table S1.

Additionally, we checked the expression of housekeeping genes (*ACTB*, *GAPDH*, and *PPIA*) and apoptosis-related genes (*BID*, *BAK1*, and *CASP9*) in each sample to investigate cell viability during this sequencing process (Additional file 3: Fig. S2). Referring PCA plots for QC (Additional file 2: Fig. S1B), the expression levels of housekeeping genes necessary for survival were low in the population of cells excluded through QC, and they also hardly expressed apoptosis-related genes. The population of cells that passed QC expressed certain levels of housekeeping genes in all E12, E16, and hatch, and they expressed low levels of apoptosis-related genes, which were similar between the different samples. Particularly in hatch samples, we checked the expression of the genes mainly mentioned in this study in the cell population excluded from QC (Additional file 4: Fig. S3), and it was verified that the QC criteria did not affect the results and only high-quality cells were selected.

After aggregation of gene-by-cell count matrices of E12, E16, and hatch, to remove cell-specific biases, cells were clustered by using the quickCluster function of the scran, version 1.16.0, R package [44]. Cell-specific size factors were computed by using the computeSumFactors function of the same package. The aggregated count matrix was normalized by dividing the raw UMI counts by the computed size factors. The normalized counts were log_2_-transformed by adding a pseudo-count of 1. Highly variable genes (HVGs) were defined as 750 genes with respect to biological variability using the decomposeVar and the getTopHVGs function of the scran package.

The k-nearest neighbor (kNN) graph was computed with FindNeighbors function of Seurat, version 4.0.1, R package [45] on the first 15 principal components (PCs) and used to compute clusters by using FindClusters function with resolution = 0.25. The 15 PCs were used to calculate uniform manifold approximation and projection (UMAP) by using RunUMAP function of the same package.

DEGs between conditions were identified using FindMarkers function of the Seurat R package. K-means clustering was performed on all DEGs using ‘cluster (version 2.1.2)’ R package and the gap statistics was used to determine the optimum number of clusters [46]. GO terms enrichment analysis was performed by using PANTHER [47] and “GO biological process complete” was selected as the annotation dataset. The test type was Fisher’s exact. Kyoto Encyclopedia of Genes and Genomes (KEGG) pathway enrichment analysis was performed by using DAVID 6.8 [48–50]. Significantly enriched GO terms and KEGG pathways were selected by using a *P*-value cutoff of 0.1. A list of genes belonging to terms associated with the cell cycle and epigenetic reprogramming was extracted from the AmiGO2 database [51]. Count matrices of E2.5 to 1 week post-hatch were further aggregated and re-normalized with scran R package, using the same methods above.

### Immunohistochemistry and histology

The procedures of testes section and immunostaining were followed by our previous report [52]. Testes of *DAZL::GFP* chickens were microscopically dissected from 3 embryos or 3 chicks at E12, E16, hatch, 4 d post-hatch, 1 week post-hatch, 2 weeks post-hatch, and 3 weeks post-hatch, respectively. The dissected samples were fixed in 4% paraformaldehyde overnight and dehydrated with a serial concentration of ethanol from 30% to 100%. They were then paraffin-embedded and sectioned (thickness, 10 μm). After deparaffinization, sections were washed three times with PBS and blocked with a blocking buffer (5% goat serum and 1% bovine serum albumin in PBS) for 1 h at room temperature. Sections were then incubated at 4 °C overnight with primary antibody. After washing three times with PBS, sections were incubated with fluorescence-conjugated secondary antibodies for 2 h at room temperature. After washing three times with PBS, sections were mounted with VECTASHIELD® Antifade Mounting Medium with DAPI (Vector Laboratories, Burlingame, CA, USA) and imaged using a confocal fluorescence microscope (Carl Zeiss Inc., Oberkocken, Germany). The primary antibodies were used at 1/200 dilution: rabbit anti-GFP (A11122, Thermo Fisher–Invitrogen), mouse anti-5-methylcytosine (ab10805, Abcam, Cambridge, United Kingdom), and rabbit anti-DAZL (ab215718, Abcam). Secondary antibodies were used at 1/200 dilution: Goat anti-Rabbit IgG Alexa Fluor 488 (A11034, Thermo Fisher–Invitrogen) and Goat anti-Mouse Alexa Fluor 594 (A11032, Thermo Fisher–Invitrogen).

For anti-5-mC immunostaining, some additional steps were performed. After deparaffinization, tissue sections were treated for 30 min in 0.5% Triton X-100 at room temperature and then treated for 10 min in 4 mol/L HCl at room temperature, followed by a blocking step. In addition, considering that HCl treatment impairs GFP, DAZL antibody staining was followed to identify germ cells. Fluorescence intensity at each time point was quantitated from three independent experiments with ImageJ (National Institutes of Health, Bethesda, MD, USA). The color channel was split, and only the germ cells chromatin in the red channel was analyzed by the ROI ‘manager tool’. In all images, the red channel, a parameter used for comparison for each sample, was taken with the same program settings.

To perform histological analysis, the testes section of *DAZL::GFP* chickens from E12, E16, and hatch were following the same ways as described above (thickness, 10 μm). After deparaffinization, sections were stained with hematoxylin and eosin using standard methods and imaged using Inverted Light Microscope (Carl Zeiss Inc).

### Preparation of DAZL::GFP cells for quantitative RT-PCR from *DAZL::GFP* chickens

To prepare samples for quantitative RT-PCR (qRT-PCR), the testes were microscopically dissected and pooled from 5 embryos at E12, 5 embryos at E16, and 8 chicks at hatch, respectively (*n* = 3, a total of 3 sample groups were prepared for each time point). The E12 samples were treated with HBSS containing 0.05% trypsin-EDTA for 10 min in shaking incubator at 37 °C and 250 r/min. E16 and hatch samples were treated with HBSS containing 0.25% trypsin-EDTA and 1 mg/mL collagenase (Collagenase type I; C0130, Sigma-Aldrich Corp., St. Louis, MO, USA) for 15 to 25 min in a shaking incubator at 37 °C and 250 r/min [6, 53], and the dispersed cells were filtered through 40 μm cell strainer (Falcon™ 352340, Fisher Scientific). Every 10 min, pipetting was conducted during incubation, and after finishing the incubation, trypsin EDTA solution was inactivated by the same volume of DMEM containing 5% FBS. The cell suspension was harvested by centrifugation (1250 r/min, 5 min), and washed with PBS. The dissociated cells from *DAZL::GFP* chickens were suspended with PBS containing 1% bovine serum albumin (BSA) and DAZL::GFP cells were collected through FACS Aria II (BD Biosciences) (Additional file 5: Fig. S4A).

### RNA isolation, RT-PCR, and quantitative RT-PCR

Total RNA samples from DAZL::GFP cells at E12, E16, and hatch were prepared using the ReliaPrep™ RNA Miniprep Systems (Promega, Madison, WI, USA), and then cDNAs were synthesized using the SuperScript III Reverse Transcription Kit (Invitrogen). RT-PCR was performed to ensure that DAZL::GFP cells were properly isolated by FACS (Additional file 5: Fig. S4B). The cDNAs were amplified by PCR using primer of *DAZL* and *GAPDH* (Additional file 6: Table S2). PCR reactions comprised 30 cycles at 95 °C for 30 s, 60 °C for 30 s, and 72 °C for 30 s. Positive-control RNA was extracted from WL PGCs cultured in accordance with our standard procedure [54]. Then, qRT-PCR was performed to examine the relative expression level of several DEGs using a StepOnePlus real-time PCR system (Applied Biosystems, Foster City, CA, USA) in triplicate. qRT-PCR reactions followed thermocycling conditions: 5 min at 95 °C followed by 40 cycles of 30 s at 95 °C, 30 s at 60 °C, and 30 s at 72 °C, and finally, at the melting temperatures. Quantification of relative gene expression was calculated using the following formula: *DC*_*t*_ = *C*_*t*_ of the target gene – *C*_*t*_ of *GAPDH*. Primer set information are listed in Additional file 6: Table S2.

### Immunocytochemistry

FACS-sorted DAZL::GFP cells in testicular cells pooled from 5 embryos or 5 chicks of E12, E16 and hatch, respectively, were fixed with 4% paraformaldehyde for 20 min. After permeabilization with 0.1% Tween-20 and 1% Triton X-100, nonspecific binding was blocked with blocking buffer (5% goat serum and 1% bovine serum albumin in PBS) for 1 h at room temperature. Cells were then incubated with mouse anti-GFP antibody (A11120, Thermo Fisher–Invitrogen), rabbit anti-trimethyl-histone H3K9 antibody (07–442, Millipore, Bedford, MA, USA), rabbit anti-acetyl-histone H3K9 antibody (ab61231, Abcam), and rabbit anti-acetyl-histone H3K14 antibody (ab82501, Abcam) at 4 °C overnight. Cells incubated in the absence of primary antibodies were used as a negative control. After extensive washing, cells were incubated with Alexa Fluor 488-conjugated goat anti-mouse IgG H&L (A11029, Invitrogen), Alexa Fluor 568-conjugated goat anti-rabbit IgG H&L (A11036; Thermo Fisher–Invitrogen), for 2 h at room temperature. After washing with PBS for three times, cells were mounted with VECTASHIELD® Antifade Mounting Medium with DAPI and imaged using a confocal fluorescence microscope (Carl Zeiss Inc). In all images, the red channel, a parameter used for comparison for each sample, was taken with the same program settings.

### Transmission electron microscopy (TEM)

*DAZL::GFP* chicken testes were microscopically extracted, one each at E12, E16, and hatch, and incubated in Karnovsky’s Fixation solution at 4 °C overnight. After washing with 0.05 mol/L sodium cacodylate buffer three times, the post-fixation step was conducted at 4 °C by adding 2% osmium tetroxide and 0.1 mol/L cacodylate buffer for 2 h. Briefly, after washing with distilled water, samples were incubated in 0.5% uranyl acetate at 4 °C overnight. After washing samples with distilled water, dehydration was followed with a serial concentration of ethanol from 30% to 100%. In the embedding step, ethanol was replaced with propylene oxide, and SPURR’s resin was serially added by increasing its concentration to 100%. Finally, samples were incubated at 70 °C overnight, and resin blocks were sectioned by Ultramicrotome (EM UC7, Leica, Wetzlar, Germany). All image was obtained by Transmission Electron Microscope (JEM1400Flash, JEOL, Akishima, Japan).

### Cell cycle analysis

WL wild-type chicken testes were extracted and pooled from 3 chicks at hatch, 4 d post-hatch, 1 week post-hatch, 2 weeks post-hatch, 3 weeks post-hatch, and 4 weeks post-hatch, respectively. They were treated with HBSS containing 0.25% trypsin-EDTA and 1 mg/mL collagenase (Collagenase type I; C0130, Sigma-Aldrich Corp., St. Louis, MO, USA) for 15 to 25 min in a shaking incubator at 37 °C and 250 r/min [6, 53], and the dispersed cells were filtered through 40 μm cell strainer (Falcon™ 352340, Fisher Scientific). Every 10 min, pipetting was conducted during incubation, and after finishing the incubation, trypsin EDTA solution was inactivated by the same volume of DMEM containing 5% FBS. The cell suspension was harvested by centrifugation (1250 r/min, 5 min), and washed with PBS. After washing with PBS once more, samples at each time point were fixed in 70% ethanol for 1 d at − 20 °C. The fixed cells were washed with PBS and incubated with blocking solution containing 5% goat serum and 1% bovine serum albumin (BSA; Sigma-Aldrich) in PBS for 1 h at 4 °C. Next, the cell aliquots were incubated with an anti-DAZL primary antibody (rabbit IgG, ab215718, Abcam) for 1 h at 4 °C. After washing three times with PBS containing 0.05% Tween 20 (PBST), the cells were incubated with donkey anti-rabbit IgG Alexa Fluor 647 (ab150075, Abcam) diluted in PBST (1:500) for 1 h at room temperature. After incubation, the cells were washed three times in PBST and dissociated in 1% BSA in PBS. For the cell cycle analysis, the isolated germ cells were treated with 10 μg/mL RNase A (Invitrogen) for 30 min at 37 °C and 50 μg/mL propidium iodide (PI; Sigma-Aldrich) for 30 min at 4 °C. The cell cycle status was analyzed by FACSCantoII (BD Biosciences), and data were analyzed using FlowJo software (Treestar, Inc., San Carlos, CA, USA). Considering that GFP, a soluble cytoplasmic protein, can leak out of cells after ethanol treatment, we used wild-type samples with DAZL antibody for this experiment only.

### Statistical analysis

Statistical analysis was performed using GraphPad Prism (GraphPad Software, CA, USA). The level of significance for all statistical tests was set at *P* < 0.05. Significant differences between groups were determined by one-way ANOVA with Tukey’s multiple comparisons. Statistical significance is ranked as * *P* < 0.05, ** *P* < 0.01, *** *P* < 0.001, and **** *P* < 0.0001.

## Results

### Localization of GFP-expressing germ cells during mitotic arrest in *DAZL::GFP* chicken testes

**Fig. 1:**
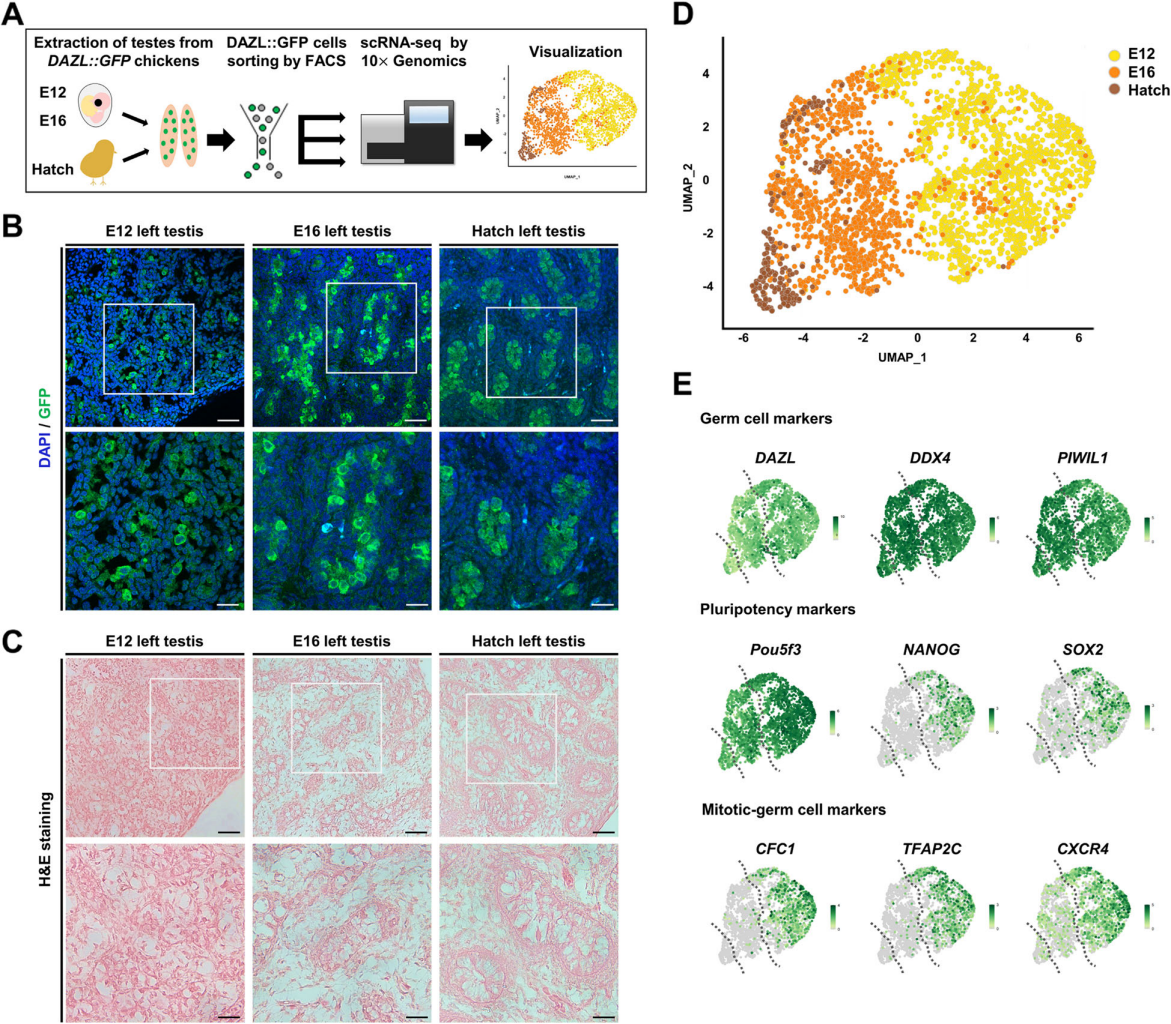
Identification of DAZL::GFP cells and single-cell transcriptome profiling in *DAZL::GFP* chickens at E12, E16, and hatch. **A** Illustration of experimental workflow. **B** Immunohistochemistry of left testes of the *DAZL::GFP* chickens at E12, E16, and hatch. Scale bars, 20 μm (upper row) and 40 μm (bottom row). **C** Hematoxylin and eosin staining of left testes of *DAZL::GFP* chickens at E12, E16, and hatch. Scale bars, 20 μm (upper row) and 40 μm (bottom row). **D** Uniform manifold approximation and projection (UMAP) plot of DAZL::GFP cells from testes of *DAZL::GFP* chickens at E12, E16, and hatch. **E** UMAP plots illustrating expression of the germ cell markers (*DAZL*, *DDX4*, and *PIWIL1*), pluripotency markers (*NANOG*, *Pou5f3*, and *SOX2*), and mitotic-germ cell markers (*CFC1*, *TFAP2C*, and *CXCR4*) at E12, E16, and hatch. The dotted lines on UMAP plots indicate the approximate division between the three samples, the right part is E12, the middle part is E16, and the lower-left part is hatch

To track and isolate germ cells at all developmental stages of chickens, we used CRISPR/Cas9-NHEJ-mediated genome editing system to insert *GFP* expression cassette into *DAZL* gene of PGC, producing *DAZL::GFP* chicken without affecting endogenous *DAZL* expression. Here, we studied mitotic arrest in male germ cells by using scRNA-seq and our established *DAZL::GFP* transgenic-chicken model. We analyzed cells at three developmental time points (E12, before mitotic arrest; E16, after onset of mitotic arrest; and hatch, in which most prospermatogonia are in G_0_/G_1_ phase) (Fig. 1A). We performed immunohistochemistry to detect DAZL::GFP cells on testes (at E12, E16, and hatch); to confirm the accuracy of germ cell tracing; and to characterize morphological signs of prospermatogonia that had entered mitotic arrest (Fig. 1B). We confirmed that DAZL::GFP cells were scattered in the testis at E12 and aggregated into the testis cords at E16. At hatch, all DAZL::GFP cells were in more clearly partitioned testis cords. In addition, aggregated germ cells, and morphological changes on the developing testis cords were further confirmed by hematoxylin and eosin staining (Fig. 1C).

### Single-cell transcriptome profiling of germ cell tracing model during mitotic arrest

To study male germ cells in mitotic arrest, we collected DAZL::GFP cells from *DAZL::GFP* chickens by fluorescence-activated cell sorting (at each time point: E12, E16, and hatch) and performed scRNA-seq on the sorted cells (Fig. 1A). After filtering out low-quality cells, we sequenced 1483 cells (at E12); 1181 cells (E16); and 241 cells (hatch), and visualized these data on the space calculated by using uniform manifold approximation and projection (UMAP) (Fig. 1D). Germ cell specific-marker genes (*DAZL*, *DDX4*, and *PIWIL1*) were strongly expressed at all time points. By contrast, both pluripotency-marker genes (*Pou5f3*, *NANOG*, and *SOX2*) and mitotic-germ cell-marker genes (*CFC1*, *TFAP2C*, and *CXCR4*) [20, 55, 56], were significantly less expressed after E12 (Fig. 1E). Given these clustering results, we investigated transcriptional dynamics during mitotic arrest at three time points.

### scRNA-seq reveals cell cycle-regulating genes and mitotic-arrested prospermatogonia

**Fig. 2:**
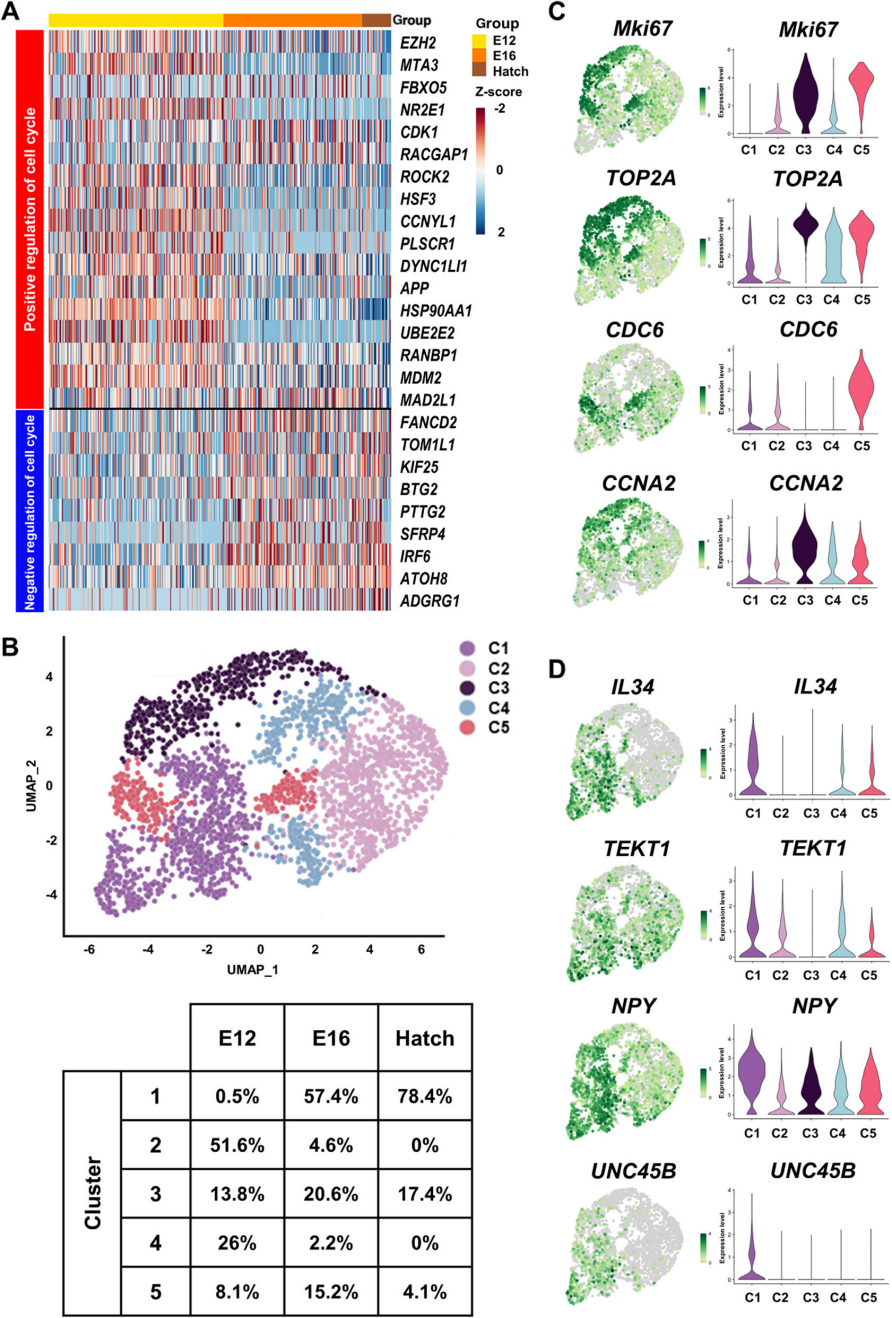
Analysis of cell cycle regulation and mitotic-arrested prospermatogonia at E12, E16, and hatch. **A** Heatmap showing expression levels of cell cycle regulation-related genes at E12, E16, and hatch. **B** UMAP plot illustrating the identified clusters after unsupervised clustering and table of each sample percentage for each cluster. **C** UMAP plots and violin plots showing expression of proliferation markers for each cluster (*Mki67*, *TOP2A*, *CDC6*, and *CCNA2*). **D** UMAP and violin plots showing the expression of C1-specific differentially expressed genes (DEGs) (*IL34*, *TEKT1*, *NPY*, and *UNC45B*). C1-C5 = Cluster1-Cluster5

To determine whether germ cells enter mitotic arrest after E12, we measured expression of cell cycle-regulating genes. We found that expression of many genes that promote cell cycle progression is lower after E12, whereas expression of those that inhibit progression is higher (Fig. 2A). Next, we performed clustering analysis at the single-cell level to confirm asynchronized mitotic arrest. We identified five clusters (C1 to C5) by applying unsupervised graph-based clustering. We visualized these clusters on the space calculated using UMAP (Fig. 2B) and then defined clusters by using proliferation-marker genes (*Mki67* and *TOP2A*), highly expressed in C3 and C5 (Fig. 2C). In particular, S phase-marker genes (*CDC6* and *CDK2* [57]) were highly expressed in C5 (Fig. 2C and Additional file 7: Fig. S5A), and G_2_/M-phase-marker genes (*CCNA2* and *CDK1* [58]) were highly expressed in C3 (Fig. 2C and Additional file 7: Fig. S5B). This indicated that C5 comprised cells in S phase, and C3 those in G_2_/M phase. Therefore, we concluded that C1 (which was the largest cluster at E16 and hatch; and was characterized by low expression levels of proliferation markers) comprised mitotic-arrested prospermatogonia. We identified C1-enriched differentially expressed genes (DEGs) highly expressed in clusters of mitotic-arrested prospermatogonia (Fig. 2D and Additional file 8: Table S3). Expression levels of spermatogonia-differentiation-associated genes (*IL34*, *TEKT1*, *NPY*, and *UNC45B* [59–62]) were significantly higher in C1 than in the other clusters. Collectively, these results demonstrated that mitotic arrest occurred asynchronously through changes in expression of cell cycle-regulating genes from E12 to hatch, consistent with our previous results showing that mitotic arrest onset occurs gradually and begins at E14.

### Initiation of mitotic arrest followed by distinct transcriptional changes in male germ cells

**Fig. 3:**
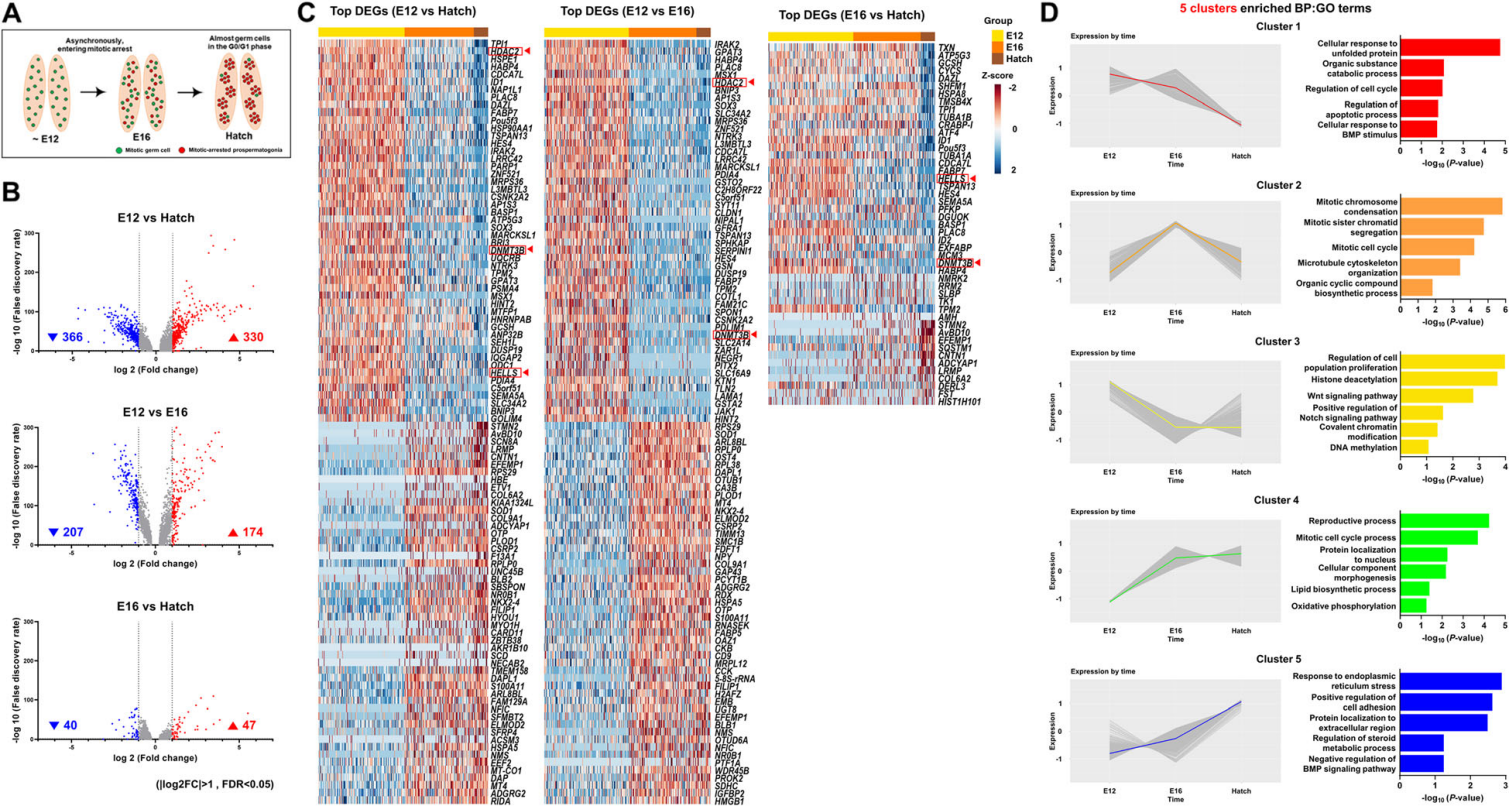
Examination of transcriptional changes in male germ cells between time points (E12, E16, and hatch). **A** Schematic representation of germ cell mitotic arrest in embryonic testes. **B** Volcano plots displaying the DEGs between groups (E12 vs. hatch, E12 vs. E16, and E16 vs. hatch). Red dots indicate log_2_ FC > 1, and blue dots indicate log_2_ FC < –1. Gray dots are DEGs with log_2_ FC between − 1 and 1, and horizontal dotted lines represent FDRs of 0.05. The numbers of red dots and blue dots are shown at the bottom of the graph. **C** Heatmaps showing top DEGs in E12 vs. hatch, E12 vs. E16, and E16 vs. hatch. Among the DEGs corresponding to |log_2_ FC| > 1 and FDR < 0.05, the 50 most upregulated and 50 most downregulated DEGs are extracted on the basis of FDR values. Fewer than 50 DEGs are shown in the heatmap of E16 vs. hatch because the heatmap includes only annotated genes. Red boxes and arrow heads indicate epigenetic-modification-related genes. **D** K-means clustering of DEGs for E12 vs. hatch, E12 vs. E16, and E16 vs. hatch, and Gene Ontology enrichment (biological process) analysis for each cluster based on K-means clustering using the PANTHER database

On the basis of our scRNA-seq results and previous cell cycle study [6], we hypothesized that mitotic arrest begins in male germ cells after E12 (Fig. 3A). To investigate transcriptional changes from E12 to hatch, we identified DEGs by comparing gene expression in germ cells at different stages: E12 vs. hatch, E12 vs. E16, and E16 vs. hatch (Fig. 3B and Additional file 9: Table S4). We found that expression levels of 330 genes were higher in hatch cells than in E12 cells, while expression levels of 366 genes were lower. Expression levels of 174 genes were higher in E16 cells than in E12 cells, while expression levels of 207 genes were lower. Finally, expression levels of 47 genes were higher in hatch cells than in E16 cells, while expression levels of 40 genes were lower. Transcriptional features in cells at the mitotically active-E12 time point differed markedly from those in cells at either hatch or E16, but transcriptional features in hatch cells were relatively similar to those in E16 cells. The expression levels of several prominent epigenetic-regulating DEGs were significantly lower in hatch cells than in E12 cells, including DEGs regulating histone deacetylation (*HDAC2*) and DNA methylation (*DNMT3B* and *HELLS*) (Fig. 3C). We observed expression of *HDAC2* and *DNMT3B* to decrease dramatically during the E12–E16 transition, and expression of *HELLS* and *DNMT3B* to decrease gradually, during the E16–hatch transition. These results suggest that the transition of chicken male germ cells to mitotic quiescence involves epigenetic regulation.

### Dynamic epigenetic regulation and biological processes during mitotic arrest

To characterize gene expression changes that occur after the onset of mitotic arrest, we conducted K-means clustering by using all DEGs identified by our comparisons (E12 vs. hatch, E12 vs. E16, and E16 vs. hatch). We identified five clusters showing temporally distinct expression patterns. For each cluster, we enriched the significant biological process GO terms by using |log_2_ FC| > 1 DEGs on the PANTHER database (Fig. 3D). Most clusters (except Cluster 5) had enriched GO terms associated with either cell cycle or proliferation. Cluster 3 (a pattern of dramatically decrease expression from E12 to E16) was enriched for GO terms associated with epigenetic modifications, such as “histone deacetylation”, “covalent chromatin modification”, and “DNA methylation”. Cluster 1 (a pattern of gradually decrease expression across three time points) was enriched for “responses to BMP” and Cluster 3 was enriched for “Wnt and Notch signaling pathways”. This result suggested that these signaling pathways are repressed in mitotic-arrested prospermatogonia (Additional file 10: Fig. S6A). We performed KEGG pathway-enrichment analysis of all DEGs without cutoff by using the DAVID database to understand signaling pathway regulation (Additional file 11: Fig. S7A). Notch signaling was enriched in both Cluster 3 and 4, suggesting that this pathway is drastically regulated at the E12–E16 transition. Receptor, ligand, effector, co-activator, and target genes mostly belonged to Cluster 3 (e.g., *NOTCH2*, *ADAM17*, *HES4*), while inhibitor and co-repressor genes mostly belonged to Cluster 4 (e.g., *DVL1*, *CTBP1*, *NCOR2*). These results indicated that Notch signaling is inhibited in mitotic-arrested prospermatogonia (Additional file 11: Fig. S7B and S7C).

Cluster 4 (a pattern of drastically increased expression between E12 and E16) was enriched for the GO terms “reproductive process” and “morphogenesis” (Additional file 10: Fig. S6B). Metabolic processes such as “lipid biosynthesis” and “oxidative phosphorylation” were also included in this cluster (Additional file 10: Fig. S6C). Cluster 5 (with gradually increased gene expression levels across the three time points) was enriched for “regulation of cell adhesion” and “protein localization to extracellular region” GO terms (Additional file 10: Fig. S6D). Cluster 5 was also enriched for “response to endoplasmic reticulum stress”, which includes *HYOU1* and *HSPA5*. Because the products of these two genes recognize and fold misfolded proteins in the endoplasmic reticulum, suggesting that quality-control process for newly synthesized proteins is activated (Additional file 10: Fig. S6E). Collectively, these results showed that genes related to epigenetic modifications were downregulated to form transcriptionally active chromatin, and that many prospermatogonia development-related genes were upregulated. Speculating that these activated biological processes were related to epigenetic regulation, we next investigated the dynamics of epigenetic regulation of germ cells during mitotic arrest in further detail.

### Global histone deacetylation and DNA demethylation during mitotic arrest

**Fig. 4:**
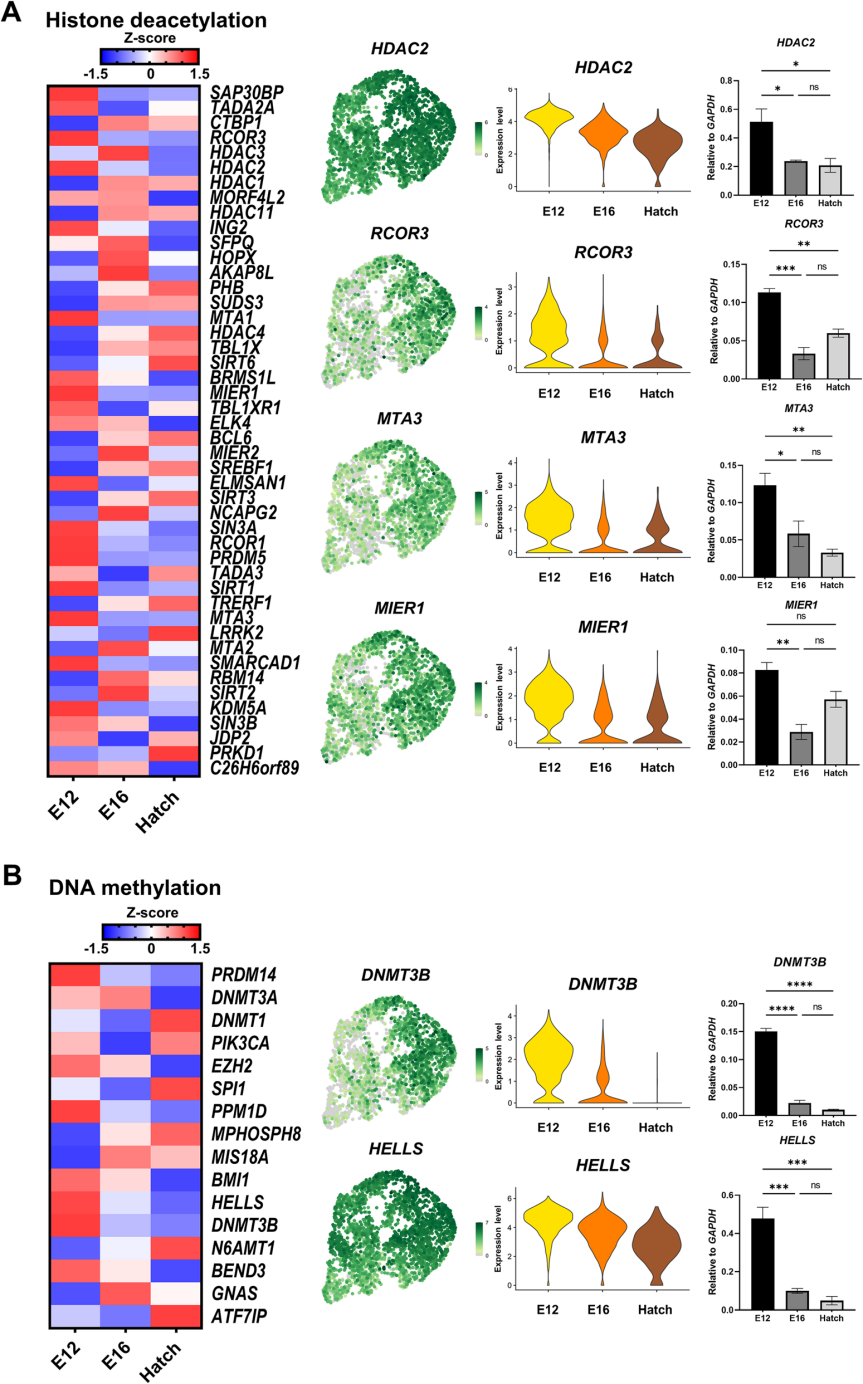
Dynamic regulation of genes associated with epigenetic regulatory processes, which especially remodel chromatin status. **A** Heatmap showing expression levels of histone-deacetylation-related genes at E12, E16, and hatch. Among these genes, DEGs (*HDAC2*, *RCOR3*, *MTA3*, and *MIER1*) are extracted, and expression levels at E12, E16, and hatch are shown using UMAP and violin plots. The mRNA expression levels (compared with *GAPDH* levels), based on quantitative RT-PCR analysis, are shown for germ cells at E12, E16, and hatch. **B** Heatmap illustrating expression of DNA-methylation-related genes at E12, E16, and hatch. Among these genes, DEGs (*DNMT3B* and *HELLS*) are extracted, and expression levels at E12, E16, and hatch are shown by using UMAP and violin plots. The mRNA expression levels (compared with *GAPDH* levels) based on quantitative RT-PCR analysis are shown for germ cells at E12, E16, and hatch. Quantitative RT-PCR data are expressed as the mean ± standard error of mean (*n* = 3). Significant differences are determined by one-way ANOVA with Tukey’s multiple comparison test. * *P* < 0.05, ** *P* < 0.01, *** *P* < 0.001, and **** *P* < 0.0001. ns = non-significant

To investigate global epigenetic reprogramming during mitotic arrest, we extracted lists of genes related to histone deacetylation and DNA methylation from the AmiGO2 database; and analyzed their expression patterns at each time point. Expression levels of several histone deacetylation-related genes (including *RCOR3*, *HDAC2*, *ING2*, *MTA1*, *BRMS1L*, *MIER1*, *SIN3A*, *RCOR1*, *SIRT1*, *MTA3*, and *KDM5A*) were markedly decreased across time points (Fig. 4A). Of these, *HDAC2* (the first class of histone deacetylases, or HDACs) and *RCOR3*, *MTA3*, and *MIER1* (HDAC-complex members) were DEGs (|log_2_ FC| > 1, FDR < 0.05). We validated the relative expression levels of genes at each time point by performing quantitative RT-PCR using DAZL::GFP cells isolated by fluorescence-activated cell sorting. We found that expression levels of histone deacetylation-related genes (relative to *GAPDH*) were consistent with the levels that we calculated from sequencing data.

In the list of genes related to DNA methylation, expression levels of *PRDM14*, *EZH2*, *PPM1D*, *BMI1*, *HELLS*, *DNMT3B*, and *BEND3* were markedly decreased at the E12 to E16 transition, followed by gradually decreased expression until Hatch (Fig. 4B). Among these genes, *DNMT3B* (which is a de novo DNA methyltransferase) and *HELLS* (which supports DNA methylation) are DEGs (|log_2_ FC| > 1, FDR < 0.05). We verified the expression levels of these genes (relative to *GAPDH*) in DAZL::GFP cells by quantitative RT-PCR, and confirmed that expression of *DNMT3B* and *HELLS* significantly decreased after E12. These results suggest that expression of genes regulating histone deacetylation and DNA methylation decreased after germ cells entered mitotic arrest. Thus, we suggest that global histone acetylation and DNA demethylation occur after the onset of mitotic arrest and are maintained until hatching.

Furthermore, we extracted a list of DNA methylation involved in gamete generation-related genes from AmiGO2. We found that expression of *TDRD5*, *MAEL*, *PRMT7*, and *TDRD1*, which suppress transposable elements, gradually increased across the three time points (Additional file 12: Fig. S8A and S8B). Therefore, although chromatin of mitotic-arrested prospermatogonia is globally demethylated and has a permissive structure, we speculate that prospermatogonia genome integrity is maintained by expression of transposon-suppressor genes.

### Mitotic-arrested prospermatogonia is regulated to permissive chromatin by histone modification

**Fig. 5:**
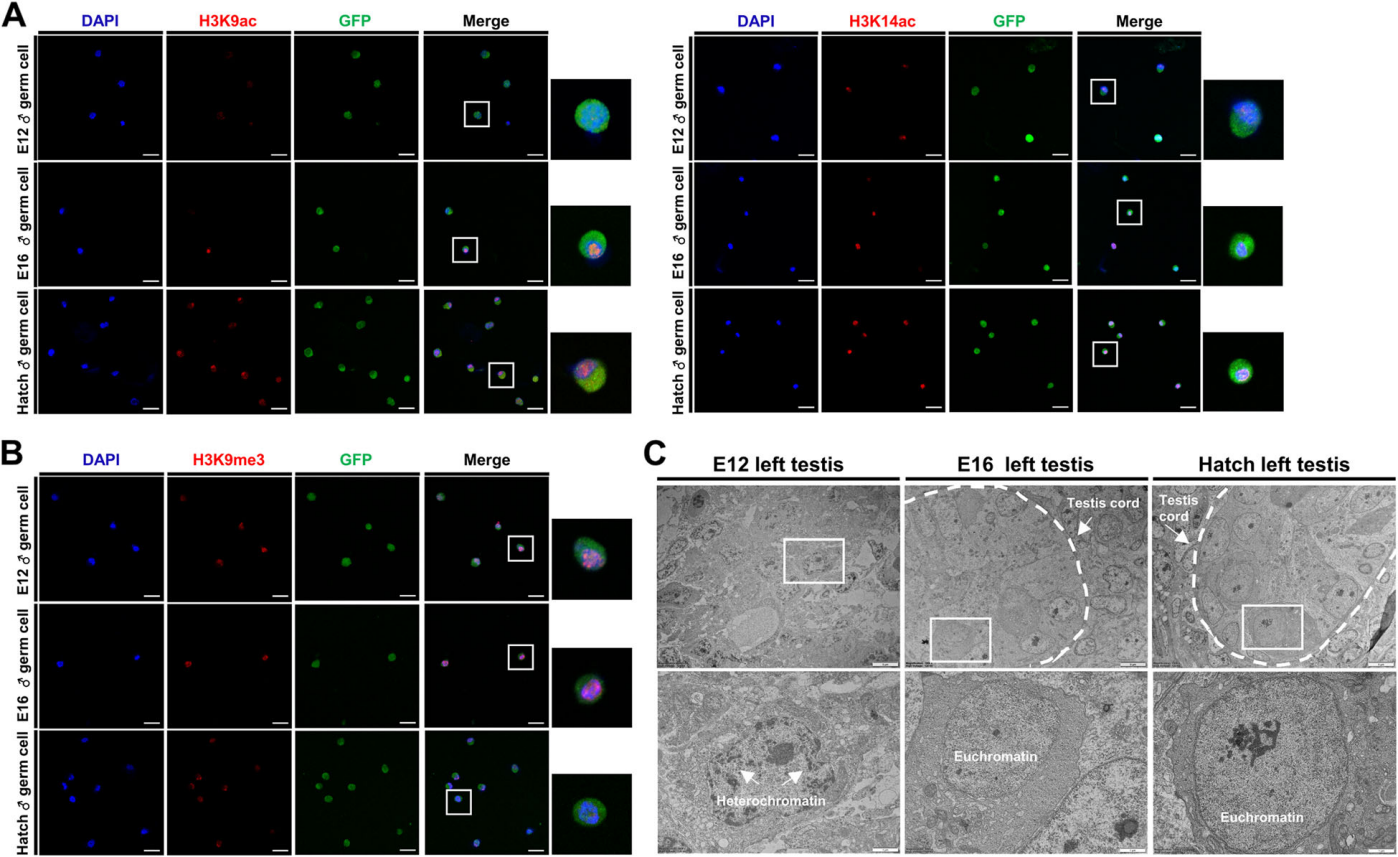
Investigation of histone modification and chromatin status of DAZL::GFP cells at E12, E16, and hatch. **A** Histone acetylation at E12, E16, and hatch. DAZL::GFP cells are stained with anti-H3K9ac and anti-H3K14ac antibodies and with DAPI. Scale bars, 20 μm. **B** Expression of heterochromatin-marker histone-H3K9 trimethylation at E12, E16, and hatch. DAZL::GFP cells are stained with anti-trimethyl-H3K9 antibody and DAPI (for nuclei). Scale bars, 20 μm. **C** Confirmation of chromatin structure of left testes germ cells at E12, E16, and hatch by transmission electron microscopy. Scale bars, 5 μm (upper row) and 1 μm (bottom row)

We measured levels of histone acetylation, a marker of transcriptionally activate genes, since we saw lower expression levels of histone deacetylases and HDAC-complex-related genes in mitotic-arrested prospermatogonia by scRNA-seq (Fig. 4A). We measured global acetylation levels of H3K9 acetylation (H3K9ac), which involved in the regulation of transcriptional activity of PGCs chromatin [63], and H3K14 acetylation (H3K14ac), which is mainly regulated by *HDAC2,* in DAZL::GFP cells by immunocytochemistry. we found that acetylation levels in DAZL::GFP cells increased after the onset of mitotic arrest (Fig. 5A). We also observed histone modifications related to transcription activity of genes at each time point. We measured heterochromatin-marker H3K9 trimethylation (H3K9me3) levels in DAZL::GFP cells. In the nucleus of DAZL::GFP cells, H3K9me3 intensity gradually decreased over time (Fig. 5B). These results suggest that mitotic-arrested prospermatogonia forms transcriptionally active chromatin structures (euchromatin form).

To determine the chromatin status of germ cells at these time points, we extracted testis at each time point to examine internal cross-sections by transmission electron microscopy (Fig. 5C). Germ cells of E12 testes contained much darkly stained heterochromatin, mostly near the nuclear envelope. Germ cells of E16 testes predominantly contained euchromatin, which is prevalent in cells actively transcribing many genes [64]. Germ cells maintained a chromatin state dominated by euchromatin even in hatch. These results were consistent with our scRNA-seq transcriptome results, suggesting that euchromatin dominates in prospermatogonia after mitotic arrest entry.

### Chicken germ cell DNA is demethylated after initiation of mitotic arrest and slowly remethylated after hatching

**Fig. 6:**
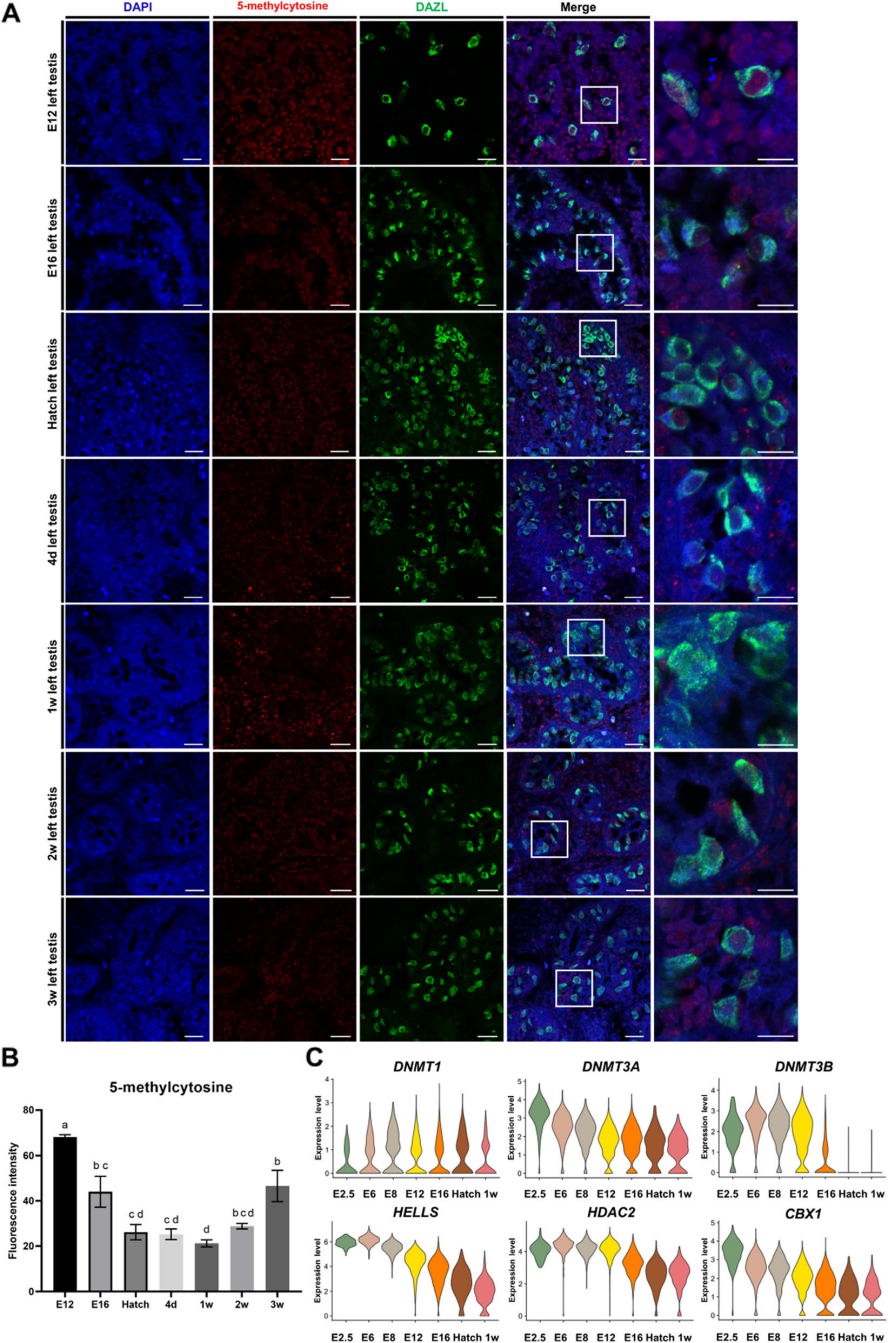
Investigation of DNA methylation of chicken male germ cells from E2.5 to after hatching. **A** Determination of global DNA methylation in left testis germ cells at E12, E16, hatch, and post-hatch (4 d, 1 week, 2 weeks, 3 weeks, and 4 weeks) by anti-5-methylcytosine (5-mC) and anti-DAZL antibody immunohistochemistry. Scale bars, 20 μm and 10 μm (enlarged images). Images are representative of those performed on *n* = 3 biologically independent animals at each developmental time point. **B** Data showing 5-mC intensity at each time point is presented as the normalized fluorescence intensity ± standard error of mean (*n* = 3/time point). ^a,b,c,d^ *P* < 0.05 between different time points using a one-way ANOVA followed by a Tukey’s post hoc test. **C** Violin plots showing expression of genes related to epigenetic reprogramming in male germ cells at E2.5 to 1 week post-hatch. d = Day post-hatch; w = Week post-hatch

We measured global DNA methylation levels of germ cells from E12 to 3 weeks post-hatch (by 5-mC and DAZL immunostaining) to confirm epigenetic reprogramming dynamics after mitotic arrest. DAZL expressing germ cell 5-mC signal was relatively strong at E12 and gradually decreased from E16 to hatch (Fig. 6A and Additional file 13: Fig. S9A). These results were consistent with our scRNA-seq transcriptome results, suggesting that DNA is globally demethylated and euchromatin dominates in prospermatogonia after mitotic arrest entry. Low 5-mC levels of germ cells were maintained at 4 d post-hatch and 1 week post-hatch. After that, DNA-methylation levels restored slowly from 2 to 3 weeks post-hatch. Further quantification observed that the intensity of 5-mC was significantly higher at 3 weeks post-hatch than at hatch and post-hatch (4 d and 1 week) (when methylation was lowest) (Fig. 6B).

Next, we confirmed the expression levels of genes involved in epigenetic regulation by scRNA-seq analysis of DAZL::GFP cells from embryonic-development stages (E2.5, E6, E8, E12, and E16); hatch; and at 1 week post-hatch. *DNMT1* (a maintenance methyltransferase) increased slightly after E2.5 and decreased slightly from E16 to 1 week post-hatch. Expression of *DNMT3A* gradually decreased from E2.5 to 1 week post-hatch. Expression of *DNMT3B*, *HELLS*, and *HDAC*2 increased from E2.5 to E6, and decreased in the E12–E16 transition and continued to be low at 1 week post-hatch. Moreover, expression of *CBX1* (a heterochromatin marker) continuously decreased over time, and we observed no significant change at 1 week post-hatch (Fig. 6C). Therefore, on the basis of DNA-methylation patterns in chicken germ cells, we propose that *DNMT3B, HELLS,* and *HDAC2* (which had identical expression patterns during development) are critical for regulating DNA methylation levels.

### Chicken germ cells continue to undergo mitotic arrest during DNA re-methylation and have unique epigenetic reprogramming patterns

**Fig. 7:**
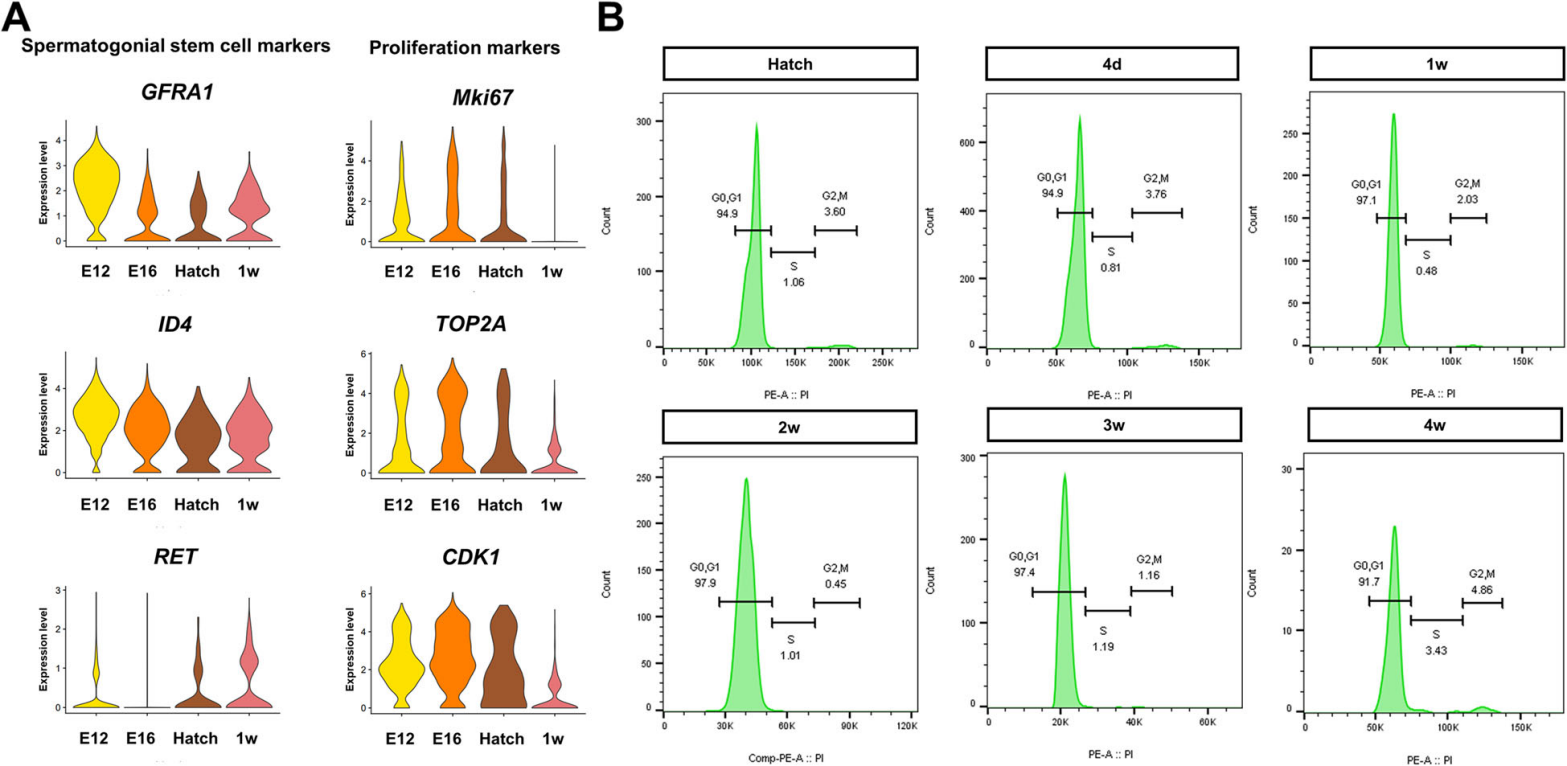
Verification of cell cycle status of DAZL-expressing germ cell after hatch. **A** Violin plots illustrate expression of both spermatogonial stem cell markers and proliferation markers at E12, E16, hatch, and 1 week post-hatch. **B** Cell cycle analysis of DAZL-expressing cells at hatch, and post-hatch (4 d, 1 week, 2 weeks, 3 weeks, and 4 weeks). d = Day post-hatch; w = Week post-hatch

We investigated the expression levels of spermatogonial stem cell-marker genes and proliferation-marker genes by scRNA-seq analysis of DAZL::GFP cells from E12 to 1 week post-hatch *DAZL::GFP* chicks. We found that expression levels of spermatogonial stem cell markers (*GFRA1*, *ID4*, and *RET*) were already increased at 1 week post-hatch than hatch, while expression levels of proliferation markers (*Mki67*, *TOP2A*, and *CDK1*) were still lower (Fig. 7A). To determine when mitosis resumes in prospermatogonia after hatching, we analyzed cell cycle progression of DAZL-expressing cells at hatch and post-hatch (4 d, 1 week, 2 weeks, 3 weeks, and 4 weeks) in White Leghorn wild-type by DAZL antibody (Fig. 7B and Additional file 14: Fig. S10). Most DAZL-expressing cells (over 90%) remained in G_0_/G_1_ phase even 4 weeks after hatching, suggesting that active mitosis had yet to resume. Therefore, we found that germ cells were not re-entered into active mitosis until re-methylation was established.

**Fig. 8:**
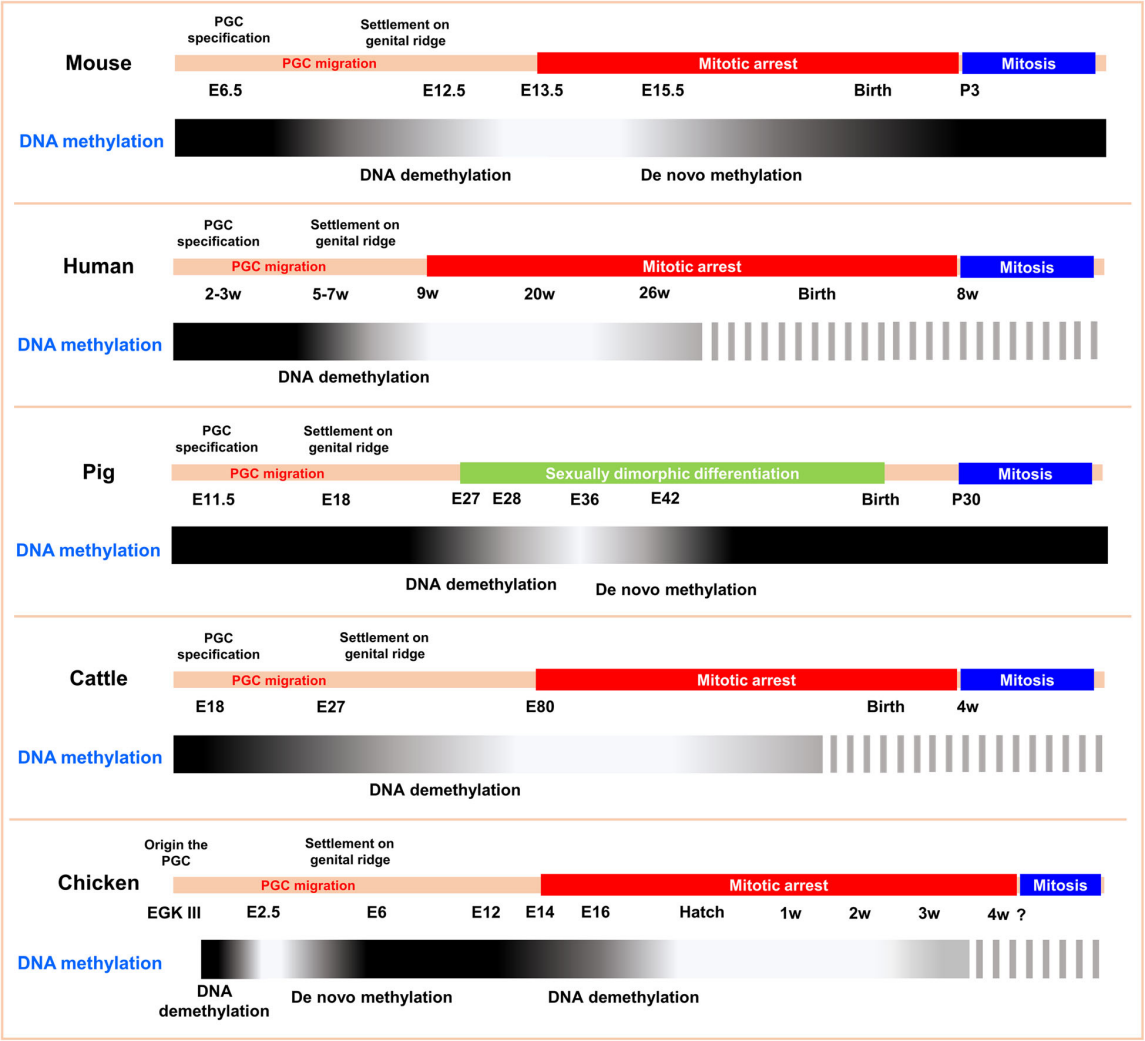
Illustration of epigenetic reprogramming and mitotic arrest in mouse, human, pig, cattle, and chicken

Taken together, in contrast to DNA methylation levels of mouse and human germ cells (which are already lowest at the onset of mitotic arrest), DNA methylation levels of chicken germ cells progressively decrease after onset of mitotic arrest and continue to decrease until hatching. Furthermore, hypomethylated state of the mitotic-arrested prospermatogonia genome persists longer in chicken compared to rapidly established re-methylation in mice and pigs, and mitotic arrest is maintained during re-methylation (Fig. 8). Therefore, we revealed that chicken male germ cells have a unique epigenetic reprogramming schedule during mitotic arrest.

## Discussion

Germ cell development toward sex-specific fates comprises a variety of complex events [2, 65]. Because germ cells that do not properly execute these events undergo apoptosis, only a few cells eventually develop into the germline [32]. In chickens, more is known about PGCs at early developmental stages (including developmental processes, in vitro cultures, and methods of transgenic-chicken production) [1] than about prospermatogonia because of the technical problem of isolating late-developmental stage germ cells. Recent advances in sequencing technology enabled single-cell resolution analyses that accelerated germ cell studies. scRNA-seq is a powerful tool to study heterogeneous and asynchronously developing germ cells (e.g., mitotic arrest and meiosis entry) in a spatiotemporal manner [18, 66–69]. In the present study, we used scRNA-seq analysis and the *DAZL::GFP* chicken germ cell tracing model to study epigenetic reprogramming in late-developmental germ cells in mitotic arrest. We found that germ cell genome methylation gradually decreased and histone acetylation levels gradually increased from the onset of mitotic arrest in chicken, unlike the process of germ cells epigenetic reprogramming in other species. In addition, low DNA methylation levels were maintained longer in chicken germ cells than in either mouse or pig germ cells, whose methylation levels are quickly re-established.

Precise epigenetic regulation is critical for differentiation and function of germ cells [26]. In our present study, we found that epigenetic reprogramming occurred after the onset of mitotic arrest, and that the structure of germ cell chromatin became more transcriptionally accessible. For differentiation of PGCs into gonocytes in mice, epigenetic reprogramming (such as DNA demethylation, PRC1-mediated inhibition, and Tet1 recruitment) is essential and increases germline reprogramming-responsive gene expression [23]. Our scRNA-seq results demonstrate that *DNMT3B* and *HELLS* expression dramatically decreases during mitotic arrest. Therefore, expression of genes such as germline reprogramming-responsive genes increases after global DNA demethylation for male-specific differentiation in chickens. Histone modifications (acetylation, phosphorylation, methylation, and ubiquitination) also regulate gene expression [70, 71]. It was reported that chicken PGC H3K9me3 levels increase during heterochromatin formation, unlike in mice exhibiting H3K27 trimethylation (H3K27me3) by polycomb protein [72]. In our study, we found that H3K9me3 levels decrease during mitotic arrest while H3K9ac and H3K14ac levels increase, suggesting that these modifications contribute to the transcription-activation state. DNA methylation and histone hypoacetylation silencing mechanisms may be connected [73]. The N-terminus of *DNMT1* binds *HDAC2* to repress transcription, which occurs only in late S phase [74]. Thus, we expect that sustained mitotic arrest is essential for chromatin structural change and maintenance.

As mitotic-arrested prospermatogonia chromatin structure becomes more permissive, upregulating biological processes are activated, including reproductive processes and intracellular development required for germ cells to differentiate into spermatogonia. In addition, we found that about 100 metabolism-related genes were upregulated by performing enriched KEGG pathway analysis. In a recent study, prospermatogonia of Retinoblastoma-1-conditional knockout mice failed to enter mitotic arrest; oxidative phosphorylation and meiosis inhibition were disrupted in these cells [7]. The authors suggested that several programming processes that occur during the quiescence period in prospermatogonia are important for subsequent normal spermatogenesis and testis development. Expression of *Cpt1a*, which regulates fatty-acid oxidation rates, increases when mouse germ cells enter mitotic arrest, and *Cpt1a*-inhibited prospermatogonia have lower histone H3K27 acetylation levels and can escape mitotic arrest [10]. Consistent with accumulating reports of the importance of metabolism-related processes during mitotic arrest, we found that various metabolism-related genes were upregulated. Also, we found that lower Notch signaling contributes to meiosis inhibition. Notch signaling regulates meiosis entry, primordial-follicle formation, and early-oocyte growth [75, 76]. Therefore, we suggest that higher levels of Notch signaling inhibitors after mitotic arrest in chickens may contribute to meiosis inhibition.

We revealed a unique epigenetic reprogramming schedule in chickens, in which male germ cells undergo genome-wide DNA demethylation after the onset of mitotic arrest and maintain low levels of DNA methylation until several weeks after hatching. We compared mitotic arrest and the epigenetic reprogramming schedules of chicken germ cells with those well reported for mouse, human, pig, and cattle (Fig. 8) [17, 28, 77, 78]. The mitotic arrest schedule of pigs is as yet unknown, but after sexually dimorphic differentiation, pig germ cell methylation is erased from E28, is minimal at E36, and increases until E42 [77]. Although mouse, human, and cattle methylation schedules differ, in all cases methylation is lowest at the onset of mitotic arrest and increases along the same timeframe. In addition, methylation of mouse and pig germ-cell genomes reached minimum levels and then recovered in 3 d (mouse) and 6 d (pig), but chicken germ cells maintained low methylation levels until after hatching and slowly recovered 3 weeks after hatch. Therefore, we propose that chickens have unique epigenetic reprogramming patterns. Except for a few species, little is known about epigenetic reprogramming during the quiescent phase that occurs specifically in male germ cells. We provided insight into this process by using our germ cell tracing model, and these findings will inform studies of epigenetic reprogramming in other species.

Unlike PGCs of mouse, human, pig, and cattle, chicken PGCs migrate through blood vessels to reach the genital ridge, where de novo methylation occurs. This difference may explain their delayed DNA demethylation schedule. However, expression of *DNMT3B* and *HDAC2* rapidly decreased during the E12–E16 transition (Fig. 6C), suggesting a correlation with onset of mitotic arrest. Further studies of mitosis re-entry in chicken are essential to identify the link between DNA methylation and mitotic arrest. We suggest several possible reasons for prolonged DNA hypomethylation in chicken prospermatogonia. DNA re-methylation in mice occurs with a rapid increase in *Dnmt3a* and *Dnmt3l* [25]. After this re-methylation, the paternal imprint is re-established [24]. However, the chicken is a nonimprinted species [79], and chicken cells do not express *Dnmt3l* [80]. In addition, the total methylation level of chicken-sperm DNA is almost half that of the mouse-sperm DNA [80]. Pig also has higher methylated sperm DNA than mouse [81]. Raddatz et al. suggested that low methylation levels of chicken sperm might be related to the absence of *Dnmt3l* [80]. Taken together, we propose that chicken germ cells may not require rapidly re-established DNA methylation during mitotic arrest, and that chicken germ cells may require lower re-methylation levels than do mouse and pig germ cells. These findings suggest that the epigenetic reprogramming schedule of chickens, unlike those of other species studied, is associated with initiating mitotic arrest.

## Conclusions

By performing scRNA-seq of *DAZL::GFP* chicken germ cells, we found that male germ cells undergo epigenetic reprogramming after the onset of mitotic arrest and adopt a permissive chromatin structure, with higher histone-acetylation levels and lower DNA-methylation levels. In addition, low methylation levels are maintained until after hatching, and methylation increases from 3 weeks post-hatch. Our study also identifies genes involved in several metabolic processes and meiosis inhibition for male germ cell-specific development. These findings are the first to dissect key events during mitotic arrest in chicken male germ cells. This information will be useful for future applications, such as high-quality germ-cell identification and in vitro spermatogenesis.

## Supplementary Information

**Additional file 1: Table S1.** Samples information of *DAZL::GFP* chicken germ cells used for scRNA-seq.

**Additional file 2: Fig. S1.** Information on quality control of the scRNA-seq. (A) Table showing thresholds of three factors for quality control of the scRNA-seq data in all samples. (B) PCA plots for quality control in samples of three time points (E12, E16, and hatch). In the QC passed plots, light blue droplets represent cells that passed through all QC criteria. nUMI = the number of unique molecular identifier; nGene = the number of detected genes; %mtDNA = proportion of the mitochondrial gene.

**Additional file 3: Fig. S2.** PCA plots showing expression of viability-related genes in process of quality control. (A) PCA plots showing the QC process in three samples. Light blue droplets included in black circles are cells that passed QC, and other red droplets indicate cells excluded from the analysis that did not pass QC criteria. (B) PCA plots showing expression of housekeeping genes (*ACTB*, *GAPDH*, and *PPIA*) in samples of three-time points (E12, E16, and hatch) in all droplets from the stage prior to exclusion from QC. As shown in above plot A, most of the droplets enclosed in the black circle represent the cell population that has passed through the QC. (C) PCA plots showing expression of apoptosis-related genes (*BID*, *BAK1*, and *CASP9*) in samples of three-time points (E12, E16, and hatch) in all droplets from the stage prior to exclusion from QC. As shown in above plot A, most of the droplets enclosed in the black circle represent the cell population that has passed through the QC.

**Additional file 4: Fig. S3.** PCA plots showing the expression of some key genes in process of quality control. (A) PCA plots showing expression of epigenetic modification-related genes (*DNMT3B*, *HELLS*, and *HDAC2*) in hatch samples. Most of the droplets included in the black circle represent the cell population that passed QC, and other droplets outside the circle represent cells that did not pass QC and were excluded from the analysis.

**Additional file 5: Fig. S4.** Isolation and verification of DAZL::GFP cells by using fluorescence-activated cell sorting (FACS) and RT-PCR. (A) FACS analysis of testicular cells from wild-type chickens (control) and *DAZL::GFP* chickens at E12, E16, and hatch. (B) *GAPDH* and *DAZL* amplicons derived from total RNA of FACS-sorted DAZL::GFP cells at E12, E16, and hatch.

**Additional file 6: Table S2.** List of primers used research.

**Additional file 7: Fig. S5.** Violin plots showing expression of cell cycle markers. (A) Violin plot showing expression of S phase marker *CDK2* for each cluster. (B) Violin plot showing expression of G_2_/M phase marker *CDK1* for each cluster.

**Additional file 8: Table S3.** List of differentially expressed genes among clusters (DEGs) (C1-C5).

**Additional file 9: Table S4.** List of DEGs between samples (E12 vs. hatch, E12 vs. E16, and E16 vs. hatch).

**Additional file 10: Fig. S6.** Violin plots showing expression of genes related to GO terms enriched in each cluster. (A) Violin plots showing expression of genes related to “cellular response to BMP stimulus”, “Wnt signaling pathway”, and “positive regulation of Notch signaling pathway” at E12, E16, and hatch. (B) Violin plots showing expression of genes related to “reproductive process” and “cellular component morphogenesis” at E12, E16, and hatch. (C) Violin plots showing expression of genes related to “lipid biosynthetic process” and “oxidative phosphorylation” at E12, E16, and hatch. (D) Violin plots showing expression of genes related to “positive regulation of cell adhesion” and “protein localization to extracellular region” at E12, E16, and hatch. (E) Violin plots showing expression of genes related to “response to endoplasmic reticulum stress protein recognition by luminal chaperones” at E12, E16, and hatch.

**Additional file 11: Fig. S7.** Kyoto Encyclopedia of Genes and Genomes (KEGG) pathway-enrichment analysis in all DEGs of each cluster. (A) KEGG pathway-enrichment analysis. (B) DEGs of Cluster 3 and Cluster 4 in the Notch signaling pathway. Yellow indicates DEGs included in cluster 3, and green indicates DEGs included in cluster 4. (C) Violin plots showing expression of genes related to Notch signaling at E12, E16, and hatch.

**Additional file 12: Fig. S8.** Dynamics of genes associated with DNA methylation involved in gamete generation. (A) Heatmap showing expression of these genes at E12, E16, and hatch. (B) UMAP and violin plot showing expression of *TDRD5*, *MAEL*, *PRMT7*, and *TDRD1* at E12, E16, and hatch.

**Additional file 13: Fig. S9.** Additional investigation of global DNA methylation of chicken male germ cells from E2.5 to after hatching. (A) Determination of global DNA methylation in left testis germ cells at E12, E16, hatch, and post-hatch (such as 4 d, 1 week, 2 weeks, 3 weeks, and 4 weeks post-hatch) by anti-5-methylcytosine (5-mC) and anti-DAZL antibody immunohistochemistry. As additional images extended in Fig. 6, images of experiments performed in three biologically independent animals each at all time points (from E12 to 3 weeks post-hatch) are shown with images showing a wider field. For each time point, the scale bars are 100 μm, 20 μm, and 10 μm from the left to right, respectively.

**Additional file 14: Fig. S10.** Gating strategy for cell cycle analysis of DAZL-expressing cells at hatch and post-hatch.

## Data Availability

The single-cell RNA sequencing data have been deposited in the SRA database under the accession code PRJNA761874. Other datasets generated during and/or analyzed during the current study are available from the corresponding authors on reasonable request.

## Abbreviations

PGC: Primordial germ cells
DEG: Differentially expressed gene
GO: Gene Ontology
PRC1: Polycomb repressive complex 1
DAZL: Deleted in azoospermia like
GFP: Green fluorescent protein
NHEJ: Nonhomologous end joining
WL: White Leghorn
QC: Quality control
UMAP: Uniform manifold approximation and projection
scRNA-seq: Single-cell RNA sequencing
DNMT: DNA methyltransferase
HELLS: Helicase, lymphoid-specific
HDAC: Histone deacetylase
5-mC: 5-methylcytosine
H3K27me3: Histone 3 lysine 27 trimethylation
H3K9ac: Histone 3 lysine 9 acetylation
H3K14ac: Histone 3 lysine 14 acetylation
