# Supplementary material for: Single-cell RNA sequencing of mitotic-arrested prospermatogonia with DAZL::GFP chickens and revealing unique epigenetic reprogramming of chickens
Source: J Anim Sci Biotechnol. 2022 Jun 6;13:64. doi: 10.1186/s40104-022-00712-4 (PMC9169296; doi:10.1186/s40104-022-00712-4)
Supplement: Supplementary file 1 — Additional file 1: Table S1. Samples information of DAZL::GFP chicken germ cells used for scRNA-seq. [file 40104_2022_712_MOESM1_ESM.pdf]

**Table S1. Samples information of DAZL::GFP chicken germ cells used for scRNA-seq.**

| Sample name | Sample details | Embryos/chicks used | $GFP^{+}$ and $PI^{-}$ cells isolated by Fluorescence-activated cell sorting | Target cells for sequencing | Cells remained after quality control |
|---|---|---|---|---|---|
| F11 | E12 male | 10 | 6,069 | 3,500 | 1,483 |
| G2 | E16 male | 10 | 5,679 | 5,000 | 1,181 |
| G4 | Hatch male | 10 | 2,069 | 1,200 | 241 |
| B7 | E2.5 male | 70-80 | 500 | 160 | 126 |
| G10 | E6 male | 35 | 1,666 | 900 | 478 |
| F9 | E8 male | 11 | 2,577 | 1,500 | 850 |
| F12 | 1w male | 3 | 1,421 | 800 | 393 |
