# Supplementary material for: Single-cell RNA sequencing of mitotic-arrested prospermatogonia with DAZL::GFP chickens and revealing unique epigenetic reprogramming of chickens
Source: J Anim Sci Biotechnol. 2022 Jun 6;13:64. doi: 10.1186/s40104-022-00712-4 (PMC9169296; doi:10.1186/s40104-022-00712-4)

Fig. S1. Information on quality control of the scRNA-seq.

A

Thresholds of three factors for quality control of scRNA-seq data.

	Condition	Sample name	Quality control threshold		
			$\log_{10}$ (nUMI)	$\log_{10}$ (nGene)	%mtDNA
Male	E12	F11	> 3.5	> 3.5	< 15
	E16	G2	> 3	> 2.5	< 15
	Hatch	G4	> 3.5	> 3	< 20
	E2.5	B7	> 3.5	> 3	< 10
	E6	G10	> 3.5	> 3	< 10
	E8	F9	> 4	> 3.5	< 15
	1w	F12	> 3.5	> 3	< 10

B

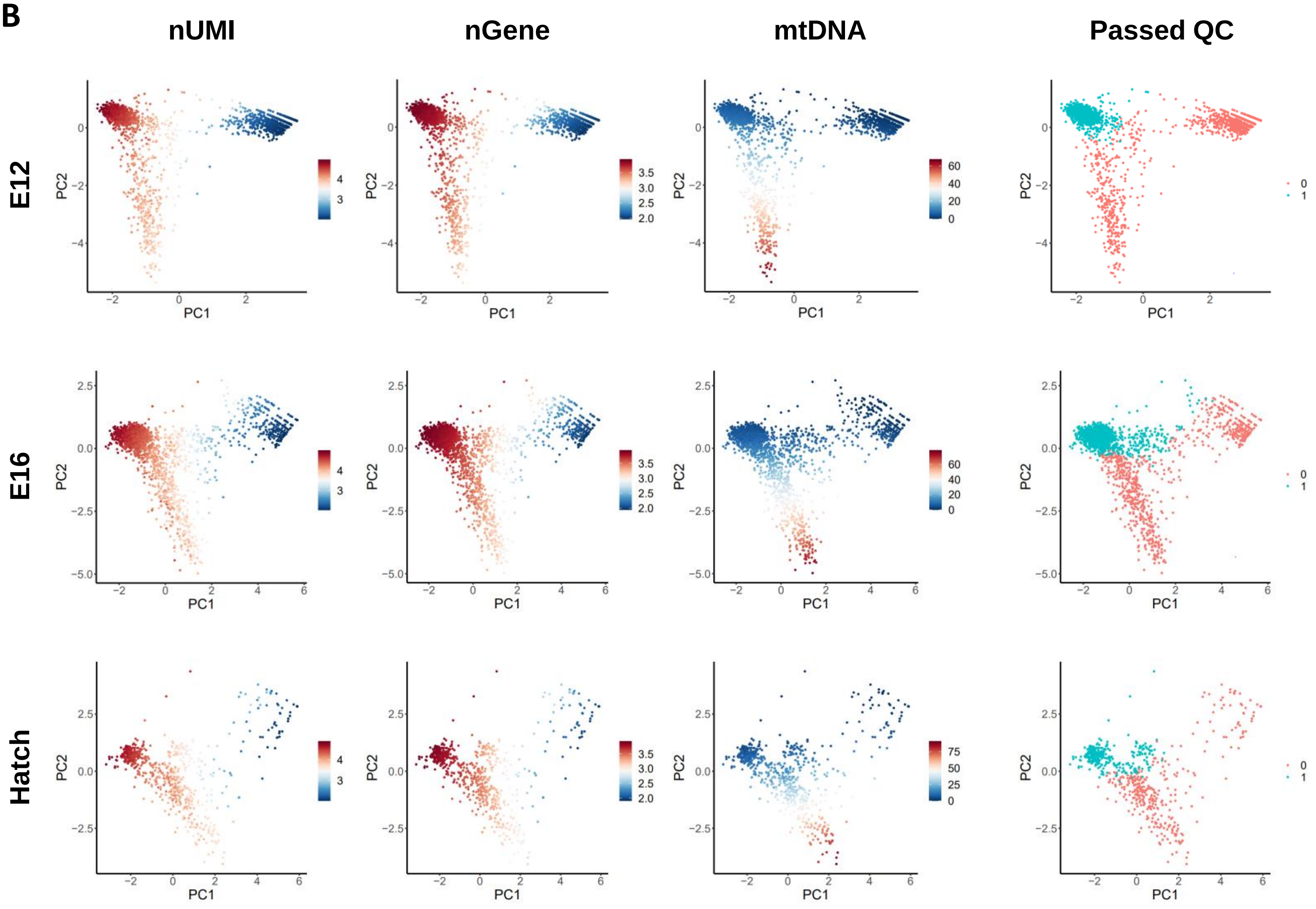

Supplement: Supplementary file 2 — Additional file 2: Fig. S1. Information on quality control of the scRNA-seq. (A) Table showing thresholds of three factors for quality control of the scRNA-seq data in all samples. (B) PCA plots for quality control in samples of three time points (E12, E16, and hatch). In the QC passed plots, light blue droplets represent cells that passed through all QC criteria. nUMI = the number of unique molecular identifier; nGene = the number of detected genes; %mtDNA = proportion of the mitochondrial gene. [file 40104_2022_712_MOESM2_ESM.pdf]
