# Supplementary figures and images for: Single-cell RNA sequencing of mitotic-arrested prospermatogonia with DAZL::GFP chickens and revealing unique epigenetic reprogramming of chickens
Source: J Anim Sci Biotechnol. 2022 Jun 6;13:64. doi: 10.1186/s40104-022-00712-4 (PMC9169296; doi:10.1186/s40104-022-00712-4)

**Fig. S2. PCA plots showing expression of viability-related genes in process of quality control.**

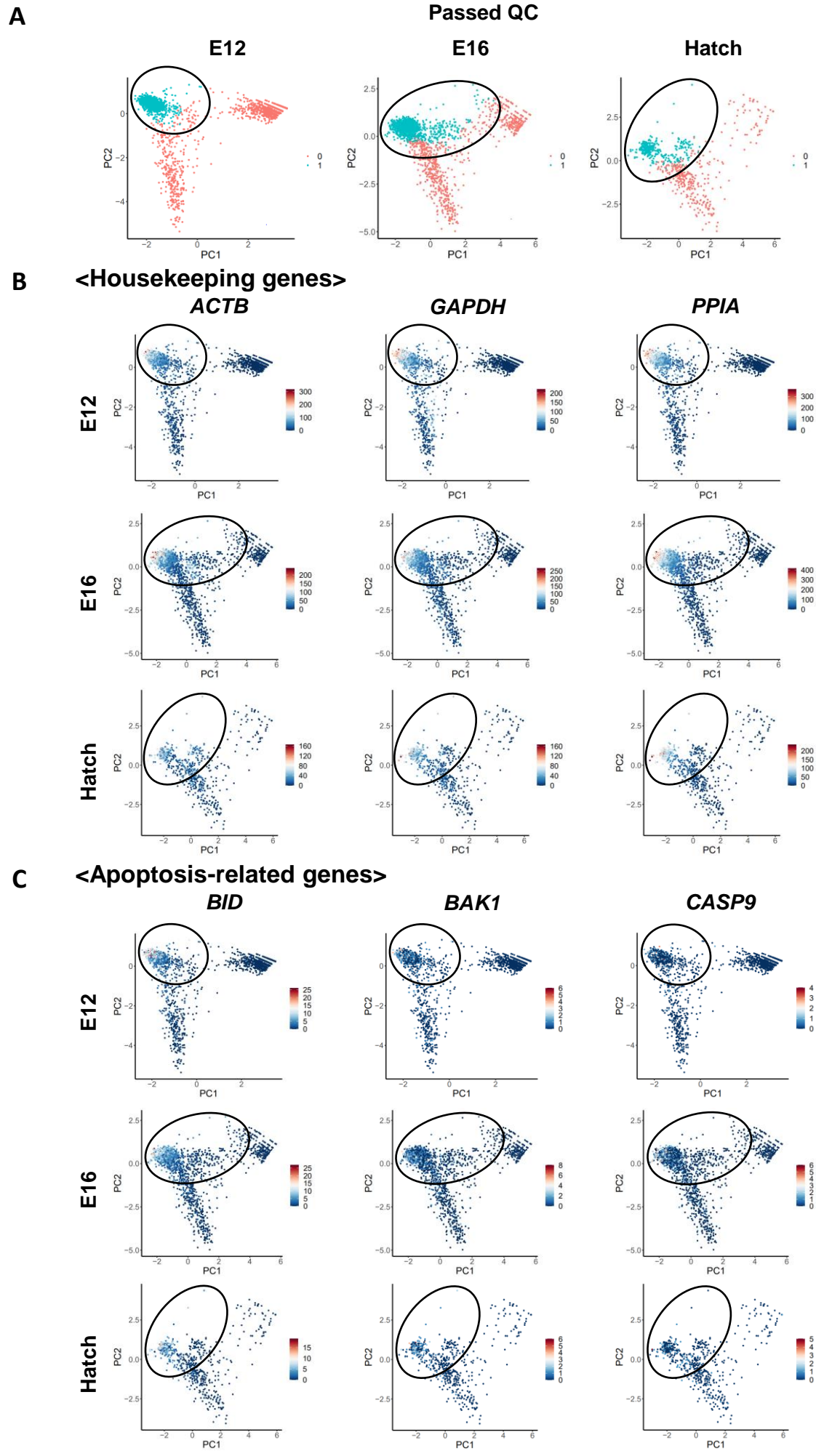

Supplement: Supplementary file 3 — Additional file 3: Fig. S2. PCA plots showing expression of viability-related genes in process of quality control. (A) PCA plots showing the QC process in three samples. Light blue droplets included in black circles are cells that passed QC, and other red droplets indicate cells excluded from the analysis that did not pass QC criteria. (B) PCA plots showing expression of housekeeping genes (ACTB, GAPDH, and PPIA) in samples of three-time points (E12, E16, and hatch) in all droplets from the stage prior to exclusion from QC. As shown in above plot A, most of the droplets enclosed in the black circle represent the cell population that has passed through the QC. (C) PCA plots showing expression of apoptosis-related genes (BID, BAK1, and CASP9) in samples of three-time points (E12, E16, and hatch) in all droplets from the stage prior to exclusion from QC. As shown in above plot A, most of the droplets enclosed in the black circle represent the cell population that has passed through the QC. [file 40104_2022_712_MOESM3_ESM.pdf]

**Fig. S3. PCA plots showing expression of some key genes in process of quality control.**

**A**

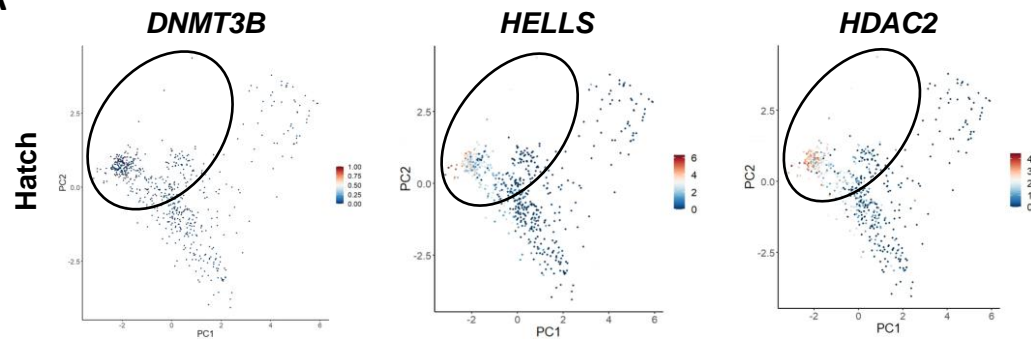

Supplement: Supplementary file 4 — Additional file 4: Fig. S3. PCA plots showing the expression of some key genes in process of quality control. (A) PCA plots showing expression of epigenetic modification-related genes (DNMT3B, HELLS, and HDAC2) in hatch samples. Most of the droplets included in the black circle represent the cell population that passed QC, and other droplets outside the circle represent cells that did not pass QC and were excluded from the analysis. [file 40104_2022_712_MOESM4_ESM.pdf]

Fig. S5. Violin plots showing expression of cell cycle markers.

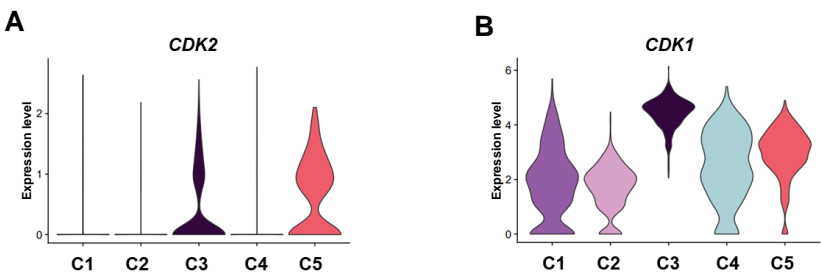

Supplement: Supplementary file 7 — Additional file 7: Fig. S5. Violin plots showing expression of cell cycle markers. (A) Violin plot showing expression of S phase marker CDK2 for each cluster. (B) Violin plot showing expression of G2/M phase marker CDK1 for each cluster. [file 40104_2022_712_MOESM7_ESM.pdf]

**Fig. S8. Dynamics of genes associated with DNA methylation involved in gamete generation.**

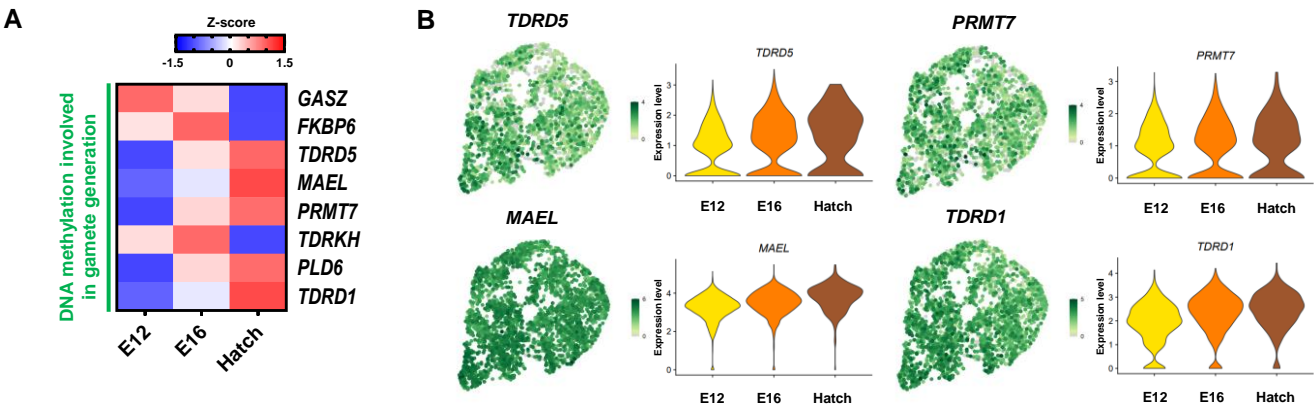

Supplement: Supplementary file 12 — Additional file 12: Fig. S8. Dynamics of genes associated with DNA methylation involved in gamete generation. (A) Heatmap showing expression of these genes at E12, E16, and hatch. (B) UMAP and violin plot showing expression of TDRD5, MAEL, PRMT7, and TDRD1 at E12, E16, and hatch. [file 40104_2022_712_MOESM12_ESM.pdf]

**Fig. S10. Gating strategy for cell cycle analysis of DAZL-expressing cells at hatch and post-hatch.**

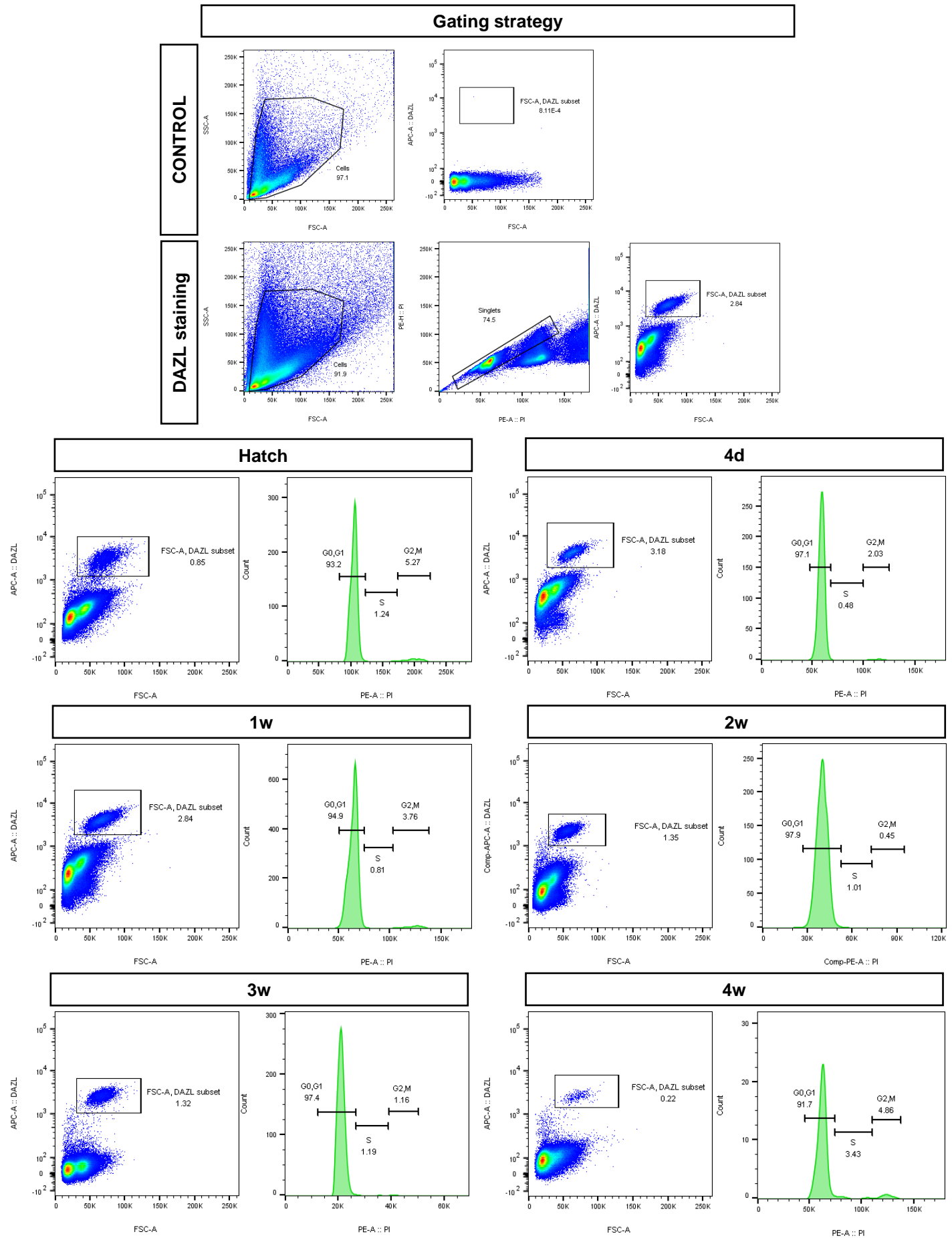

Supplement: Supplementary file 14 — Additional file 14: Fig. S10. Gating strategy for cell cycle analysis of DAZL-expressing cells at hatch and post-hatch. [file 40104_2022_712_MOESM14_ESM.pdf]
