# Supplementary material for: Single-cell RNA sequencing of mitotic-arrested prospermatogonia with DAZL::GFP chickens and revealing unique epigenetic reprogramming of chickens
Source: J Anim Sci Biotechnol. 2022 Jun 6;13:64. doi: 10.1186/s40104-022-00712-4 (PMC9169296; doi:10.1186/s40104-022-00712-4)

Fig. S4. Isolation and verification of DAZL::GFP cells by using fluorescence-activated cell sorting (FACS) and RT-PCR.

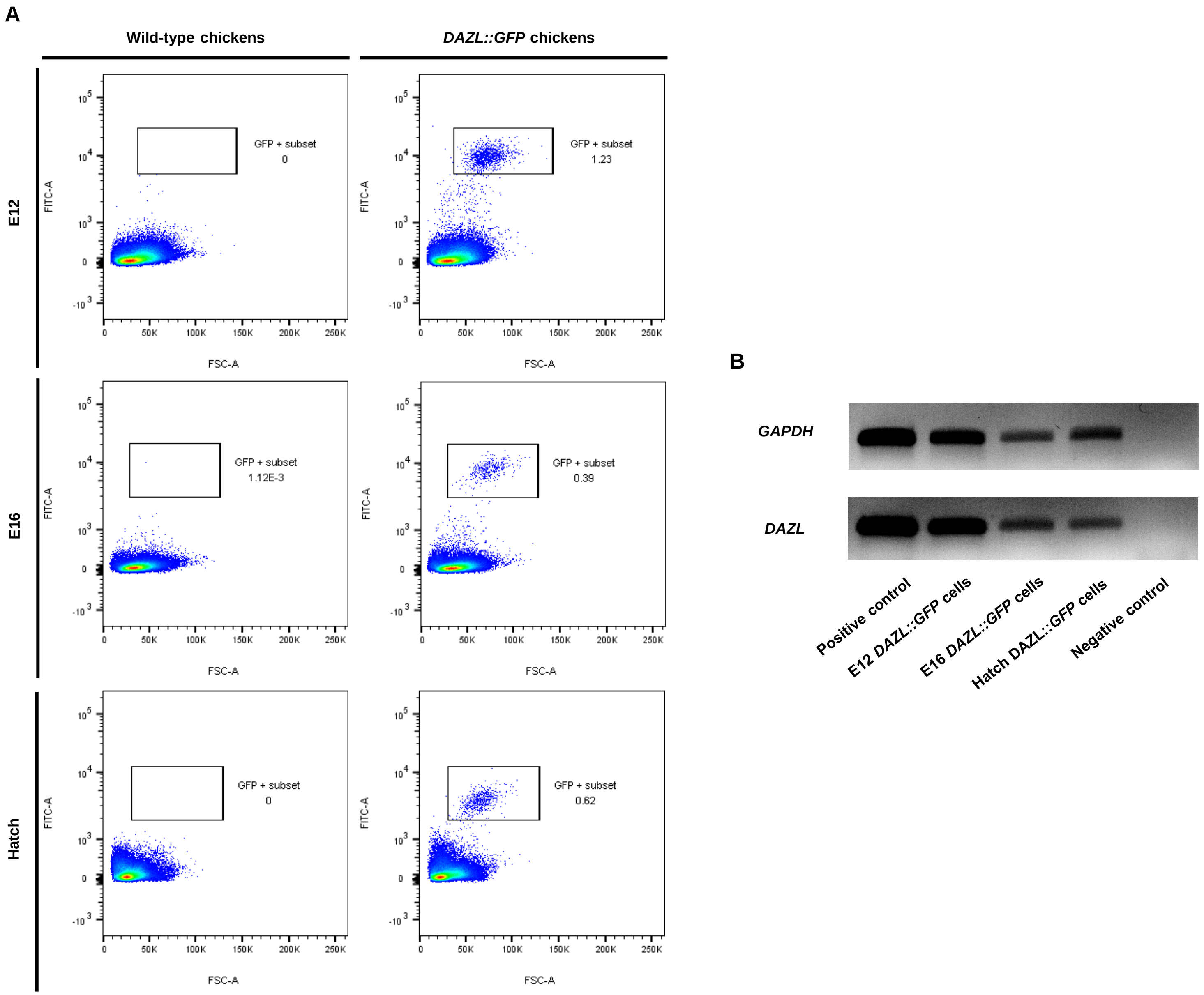

Supplement: Supplementary file 5 — Additional file 5: Fig. S4. Isolation and verification of DAZL::GFP cells by using fluorescence-activated cell sorting (FACS) and RT-PCR. (A) FACS analysis of testicular cells from wild-type chickens (control) and DAZL::GFP chickens at E12, E16, and hatch. (B) GAPDH and DAZL amplicons derived from total RNA of FACS-sorted DAZL::GFP cells at E12, E16, and hatch. [file 40104_2022_712_MOESM5_ESM.pdf]
