# Supplementary material for: Single-cell RNA sequencing of mitotic-arrested prospermatogonia with DAZL::GFP chickens and revealing unique epigenetic reprogramming of chickens
Source: J Anim Sci Biotechnol. 2022 Jun 6;13:64. doi: 10.1186/s40104-022-00712-4 (PMC9169296; doi:10.1186/s40104-022-00712-4)
Supplement: Supplementary file 6 — Additional file 6: Table S2. List of primers used research. [file 40104_2022_712_MOESM6_ESM.pdf]

Table S2. List of primers used research.

Gene symbol	Description		Primer sequence (5'→3')	Usage
GAPDH	glyceraldehyde-3-phosphate dehydrogenase	F	GGT GGT GCT AAG CGT GTT AT	RT-PCR, qRT-PCR
		R	ACC TCT GCC ATC TCT CCA CA	
DAZL	deleted in azoospermia like	F	CAA CTA TCA GGC TCC ACC AC	RT-PCR
		R	CTC AGA CGG TTT TCA GGG TT	
HDAC2	histone deacetylase 2	F	TCG AGC ATC TGA TAA GCG CA	qRT-PCR
		R	TCC TTT GGT GTC CGC CTT TT	
RCOR3	REST corepressor 3	F	CTG TAG TCC CAA TGC AGC CA	qRT-PCR
		R	AAG CTG CTC TTC TGT GGT CC	
MTA3	metastasis associated 1 family member 3	F	ATA GCC CCC TTA CAG ACC GT	qRT-PCR
		R	ACA CTG ATG GCA CTG CTC AA	
MIER1	MIER1 transcriptional regulator	F	TGG ACC GCC TCC TAG ATG AA	qRT-PCR
		R	TGC CAC CAG TAG GTC CAT TG	
DNMT3B	DNA methyltransferase 3 beta	F	GCT CGT CAG CCA AAG CAA AA	qRT-PCR
		R	AGC ACT CTG ATG GGT CTC CT	
HELLS	helicase, lymphoid specific	F	AGG AGT CCC TGG TCC CTT TT	qRT-PCR
		R	TTC AGT GAT CCT TGT CGC CC	
