# Supplementary material for: Single-cell RNA sequencing of mitotic-arrested prospermatogonia with DAZL::GFP chickens and revealing unique epigenetic reprogramming of chickens
Source: J Anim Sci Biotechnol. 2022 Jun 6;13:64. doi: 10.1186/s40104-022-00712-4 (PMC9169296; doi:10.1186/s40104-022-00712-4)

Fig. S6. Violin plots showing expression of genes related to GO terms enriched in each cluster.

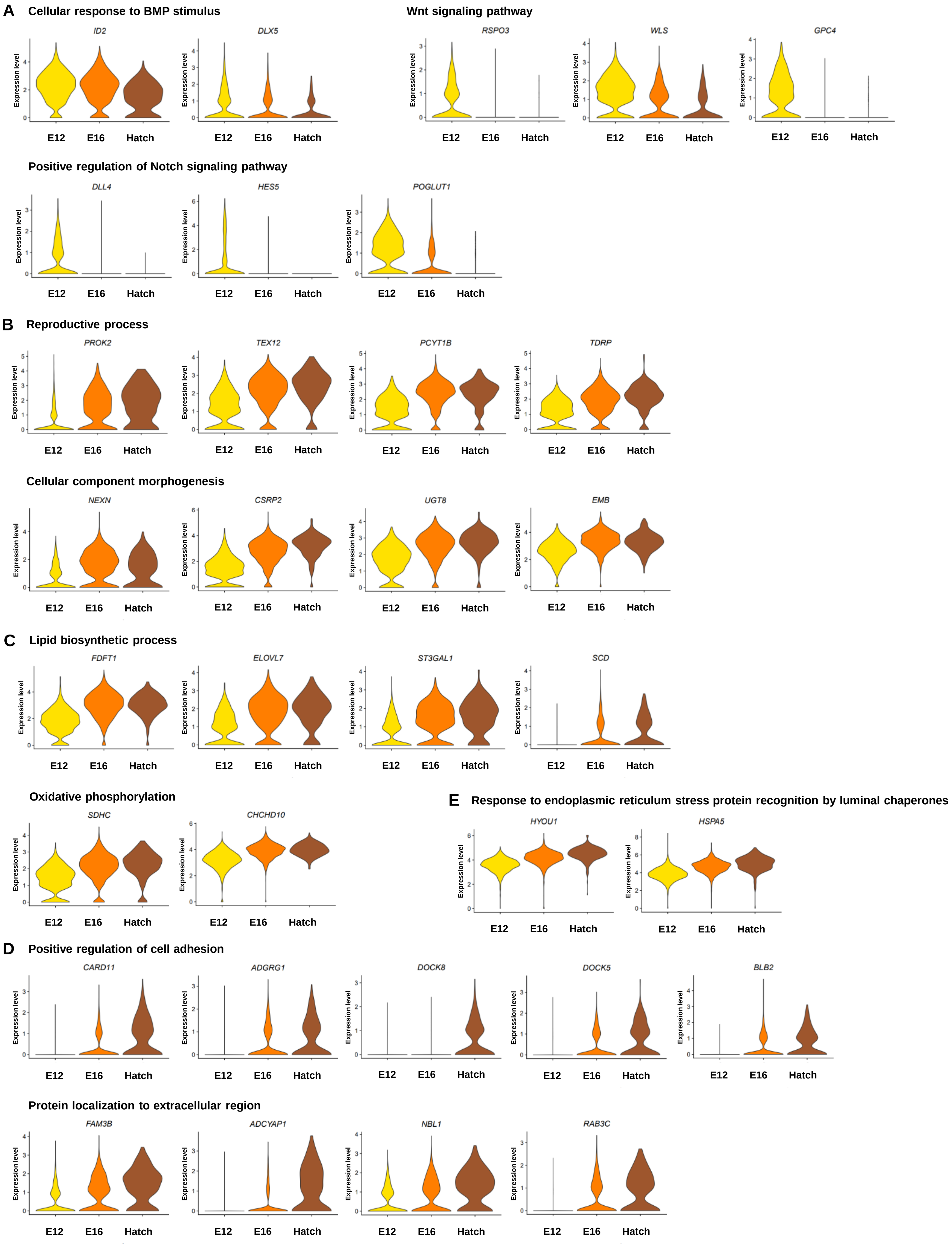

Supplement: Supplementary file 10 — Additional file 10: Fig. S6. Violin plots showing expression of genes related to GO terms enriched in each cluster. (A) Violin plots showing expression of genes related to “cellular response to BMP stimulus”, “Wnt signaling pathway”, and “positive regulation of Notch signaling pathway” at E12, E16, and hatch. (B) Violin plots showing expression of genes related to “reproductive process” and “cellular component morphogenesis” at E12, E16, and hatch. (C) Violin plots showing expression of genes related to “lipid biosynthetic process” and “oxidative phosphorylation” at E12, E16, and hatch. (D) Violin plots showing expression of genes related to “positive regulation of cell adhesion” and “protein localization to extracellular region” at E12, E16, and hatch. (E) Violin plots showing expression of genes related to “response to endoplasmic reticulum stress protein recognition by luminal chaperones” at E12, E16, and hatch. [file 40104_2022_712_MOESM10_ESM.pdf]
