# Supplementary material for: Single-cell RNA sequencing of mitotic-arrested prospermatogonia with DAZL::GFP chickens and revealing unique epigenetic reprogramming of chickens
Source: J Anim Sci Biotechnol. 2022 Jun 6;13:64. doi: 10.1186/s40104-022-00712-4 (PMC9169296; doi:10.1186/s40104-022-00712-4)

Fig. S7. Kyoto Encyclopedia of Genes and Genomes (KEGG) pathway-enrichment analysis in all DEGs of each cluster.

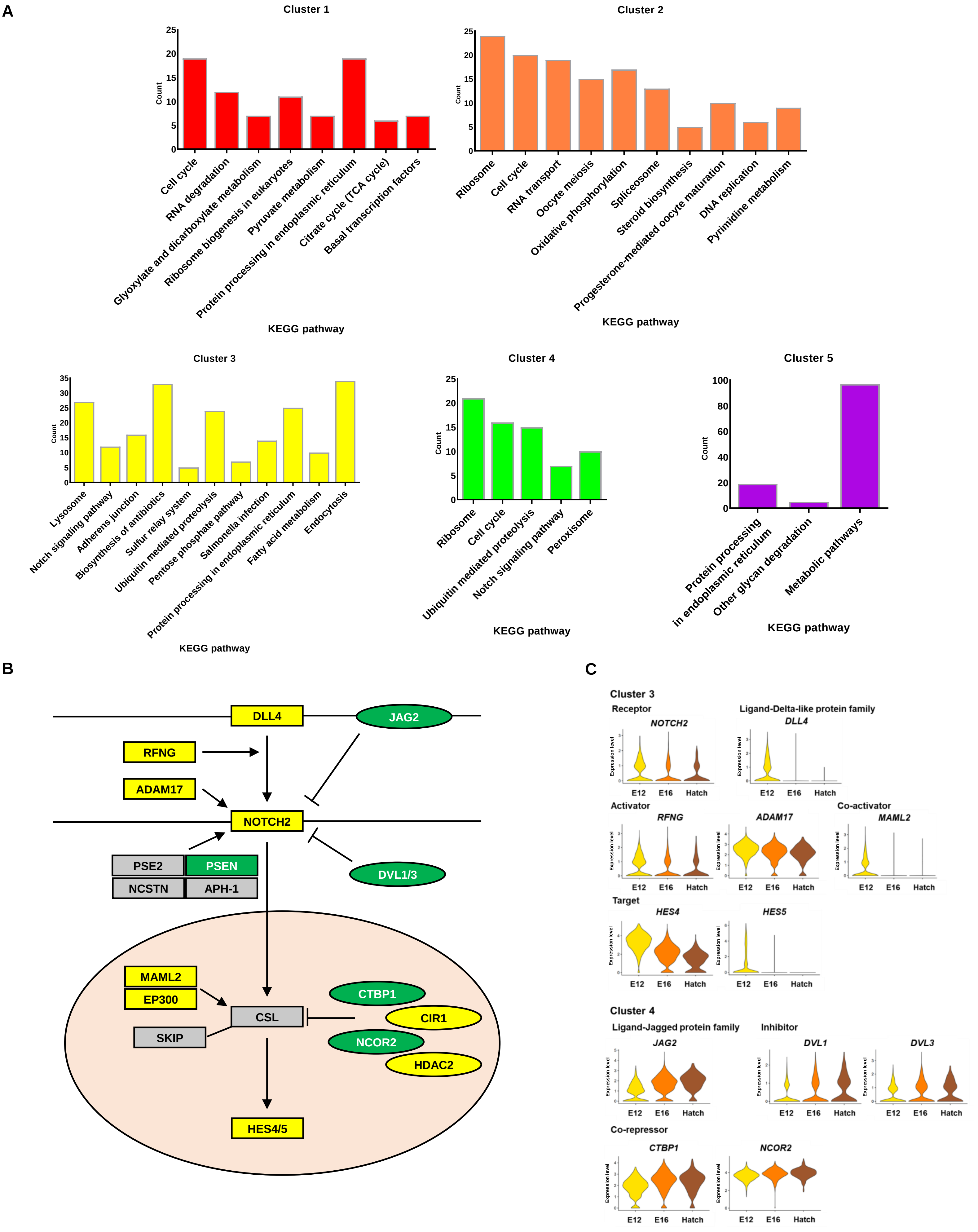

Supplement: Supplementary file 11 — Additional file 11: Fig. S7. Kyoto Encyclopedia of Genes and Genomes (KEGG) pathway-enrichment analysis in all DEGs of each cluster. (A) KEGG pathway-enrichment analysis. (B) DEGs of Cluster 3 and Cluster 4 in the Notch signaling pathway. Yellow indicates DEGs included in cluster 3, and green indicates DEGs included in cluster 4. (C) Violin plots showing expression of genes related to Notch signaling at E12, E16, and hatch. [file 40104_2022_712_MOESM11_ESM.pdf]
