# Supplementary material for: Single-cell RNA sequencing of mitotic-arrested prospermatogonia with DAZL::GFP chickens and revealing unique epigenetic reprogramming of chickens
Source: J Anim Sci Biotechnol. 2022 Jun 6;13:64. doi: 10.1186/s40104-022-00712-4 (PMC9169296; doi:10.1186/s40104-022-00712-4)

Fig. S9. Additional investigation of global DNA methylation of chicken male germ cells from E2.5 to after hatching.

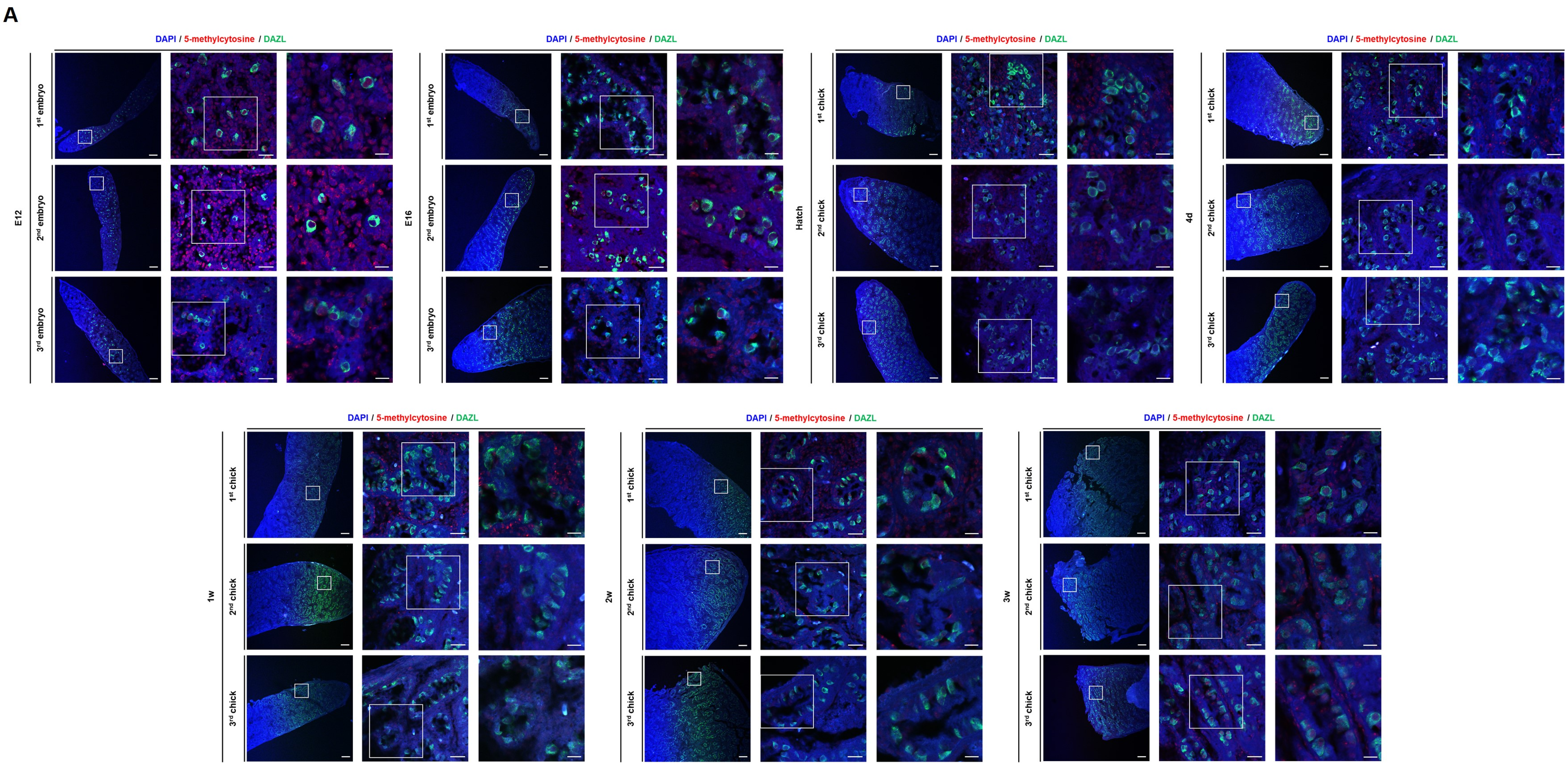

Supplement: Supplementary file 13 — Additional file 13: Fig. S9. Additional investigation of global DNA methylation of chicken male germ cells from E2.5 to after hatching. (A) Determination of global DNA methylation in left testis germ cells at E12, E16, hatch, and post-hatch (such as 4 d, 1 week, 2 weeks, 3 weeks, and 4 weeks post-hatch) by anti-5-methylcytosine (5-mC) and anti-DAZL antibody immunohistochemistry. As additional images extended in Fig. 6, images of experiments performed in three biologically independent animals each at all time points (from E12 to 3 weeks post-hatch) are shown with images showing a wider field. For each time point, the scale bars are 100 μm, 20 μm, and 10 μm from the left to right, respectively. [file 40104_2022_712_MOESM13_ESM.pdf]
